# Controlling Electronic Coupling of Acene Chromophores on Quantum Dot Surfaces through Variable-Concentration Ligand Exchange

**DOI:** 10.1021/acsnano.3c03498

**Published:** 2023-07-26

**Authors:** Marissa S. Martinez, Michelle A. Nolen, Nicholas F. Pompetti, Lee J. Richter, Carrie A. Farberow, Justin C. Johnson, Matthew C. Beard

**Affiliations:** † Chemistry & Nanoscience Center, National Renewable Energy Laboratory, Golden, Colorado 80401, United States; ‡ Department of Chemical and Biological Engineering, Colorado School of Mines, Golden, Colorado 80401, United States; § Department of Chemistry, University of Colorado Boulder, Boulder, Colorado 80309, United States; ∥ Materials Science and Engineering Division, National Institute of Standards and Technology, Gaithersburg, Maryland 20899, United States; ⊥ Catalytic Carbon Transformation & Scale-Up Center, National Renewable Energy Laboratory, Golden, Colorado 80401, United States

**Keywords:** quantum dots, electronic hybridization, QD films, ligand orientation, lead sulfide

## Abstract

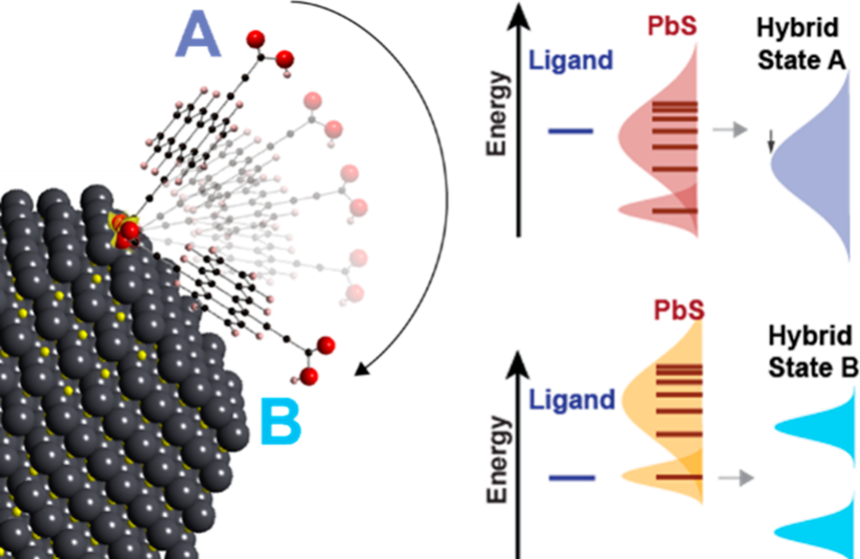

Controlling the binding of functional organic molecules on quantum dot (QD) surfaces and the resulting ligand/QD interfacial structure determines the resulting organic–inorganic hybrid behavior. In this study, we vary the binding of tetracenedicarboxylate ligands bound to PbS QDs cast in thin films by performing solid-state ligand exchange of as-produced bound oleate ligands. We employ comprehensive Fourier-transform infrared (FTIR) analysis coupled with ultraviolet–visible (UV–vis) spectrophotometric measurements, transient absorption, and Density Functional Theory (DFT) simulations to study the QD/ligand surface structure and resulting optoelectronic properties. We find that there are three primary QD/diacid structures, each with a distinct binding mode dictated by the QD–ligand and ligand–ligand intermolecular and steric interactions. They can be accessed nearly independently of one another via different input ligand concentrations. Low concentrations produce mixed oleate/tetracene ligand structures where the tetracene carboxylates tilt toward QD surfaces. Intermediate concentrations produce mixed oleate/tetracene ligand structures with ligand–ligand interactions through intramolecular hydrogen bonding with the ligands perpendicular to the QD surface and weaker QD/ligand electronic interactions. High concentrations result in full ligand exchange, and the ligands tilt toward the surface while the QD film compacts. When the tetracene ligands tilt or lie flat on the QD surface, the benzene ring π-system interacts strongly with the p-orbitals at the PbS surface and produces strong QD–ligand interactions evidenced through QD/ligand state mixing, with a coupling energy of ≈700 meV.

Hybrid QD organic/inorganic hybrid systems, where the organic component is chemically bound to the inorganic nanocrystal surface and can electronically interact with the core-QD electronic states, are interesting for controlling energy flow in a variety of applications including solar energy conversion, light emission, biomedical diagnostics or therapies, photocatalysis, etc. For solar energy harvesting, QDs alone have the advantage of being photorobust and size tunable and have the potential of boosting the single-junction solar energy conversion efficiency via multiple exciton generation (MEG) up to 44%^1^ compared to 33%^2^ in the absence of carrier multiplication. Combining inorganic QDs with organic photoactive ligands, i.e., molecular dyes, provides greater tunability and thus control of the resulting hybrid properties and generates alternate strategies for high efficiencies. Two common schemes studied for solar energy conversion with QD/dye systems are singlet-fission (SF)-based carrier multiplication and photon upconversion. In the former, a SF chromophore generates triplet excitons that are transferred to the QD where they can be further harvested, and in the latter, excitons generated in the QD transfer to the molecular chromophore, which can then undergo upconversion to emit a higher energy photon.^3−7^ In addition to those approaches, the inorganic core can photosensitize surface bound SF molecules when light is absorbed within the QD and the energy is subsequently transferred to the singlet (S_1_) states of the surface-attached SF molecules to trigger the SF process.^8,9^ The reverse can also be used to enhance or sensitize the MEG process that occurs in the QDs if light is first absorbed into surface-attached molecules and the energy is subsequently transferred to the QDs with sufficient excess energy to drive the MEG process.^10^ These are just two examples of beneficial energy flow between the inorganic QD core and surface-attached organic molecules. There is an abundance of studies on the excited state energy flow in QD–ligand systems in colloidal solutions: for instance, triplet energy transfer from carboxylic acid functionalized tetracene (TIPS-Tc-COOH) was reported to proceed in about 100 ns,^11^ and it was shown that the energy can be cycled back and forth between PbS QDs and TIPS-Tc-COOH ligands,^12^ depending on the energy alignment of the QD excitonic state with that of the molecular triplet energy levels. Researchers have shown that triplet transfer from ligands to QDs can be moderated by an intermediate charge transfer state, and this can slow the energy transfer rate.^13,14^ There are fewer studies of such phenomena in QD thin films where the energy flow can likely be better exploited, and here, we are motivated to understand and control the fundamental interactions between derivatized tetracene ligands on PbS QD surfaces as thin QD films. We are particularly interested in how the organic and inorganic building blocks interact to produce a particular physical behavior.

Broadly, QD–ligand coupling occurs through the direct bonding of the ligand via the anchoring group and can vary depending on the QD composition, size, and morphology as well as the ligand identity and its internal structure. Carboxylate-functionalized aromatic ligands exhibit QD–ligand interactions that increase the absorption cross section, shift the band-edge potentials, and shift the energy levels of the QD core.^15−21^ These electronic impacts are made possible by the interaction of the frontier molecular orbitals with those of the QD band-edge states. Ligand–ligand interactions^22−25^ on the QD surfaces can also impact the ligand exchange process and be utilized to construct ligand surface structures due to dipole–dipole intramolecular interactions.^23^ For example, when there is a sufficient balance of ligand–ligand and QD–ligand interactions, asymmetric, phase-separated ligand structures can be fabricated, i.e., Janus-ligand shells, and the induced asymmetry of the QD/ligand system can be used to produce functional optoelectronic systems.^23,24,26,27^

In this study, we functionalized PbS QDs with the dicarboxylic acid tetracene derivative Tc(Ac-COOH)_2_ (see Figure 1). The acetylene spacer group between the acene core and the carboxylic acid anchors, as well as the overall rigidity of the molecule, predispose it to form π-stacked and hydrogen-bonded aggregates in solution. Although the solution dynamics of the diacid are not explored in this study, the intramolecular ligand interactions that occur in solution can also occur when bound to QD surfaces and such interactions can be exploited to design specific QD/ligand structures. The diacid functionalization introduces the possibility of several different binding motifs, e.g., a bidentate surface structure, where one diacid ligand could lie flat on the QD surface and be tightly bound to a single nanocrystal, or the diacid could bridge/cross-link two adjacent nanocrystals. We find that micromolar quantities of the diacid added to a stable colloidal suspension of PbS QDs terminated with oleate ligands (PbS/OA) results in immediate flocculation of the QDs, even though the ligand solution and QD colloidal solution are both stable in the same solvent system. This is in stark contrast to the situation where the monoacid version of the ligand is employed. In that case, stable colloidal dispersions can be prepared at high ligand concentrations.^12^ This observation suggests that the QDs aggregate upon ligand exchange either through cross-linking of the QDs via the diacid functionality or via tightly bound ligands lying flat on the QD surface, reducing the ability of the ligands to interact with the solvent. Thus, in order to study the diacid ligand exchange reactions, we developed a solid-state ligand exchange procedure. We found that the chemical and electronic properties of the tetracene dicarboxylic acids bound to the QD surfaces have a concentration dependence, which we reveal through steady-state optical and structural studies of the resulting PbS QD films.

**Figure 1:**
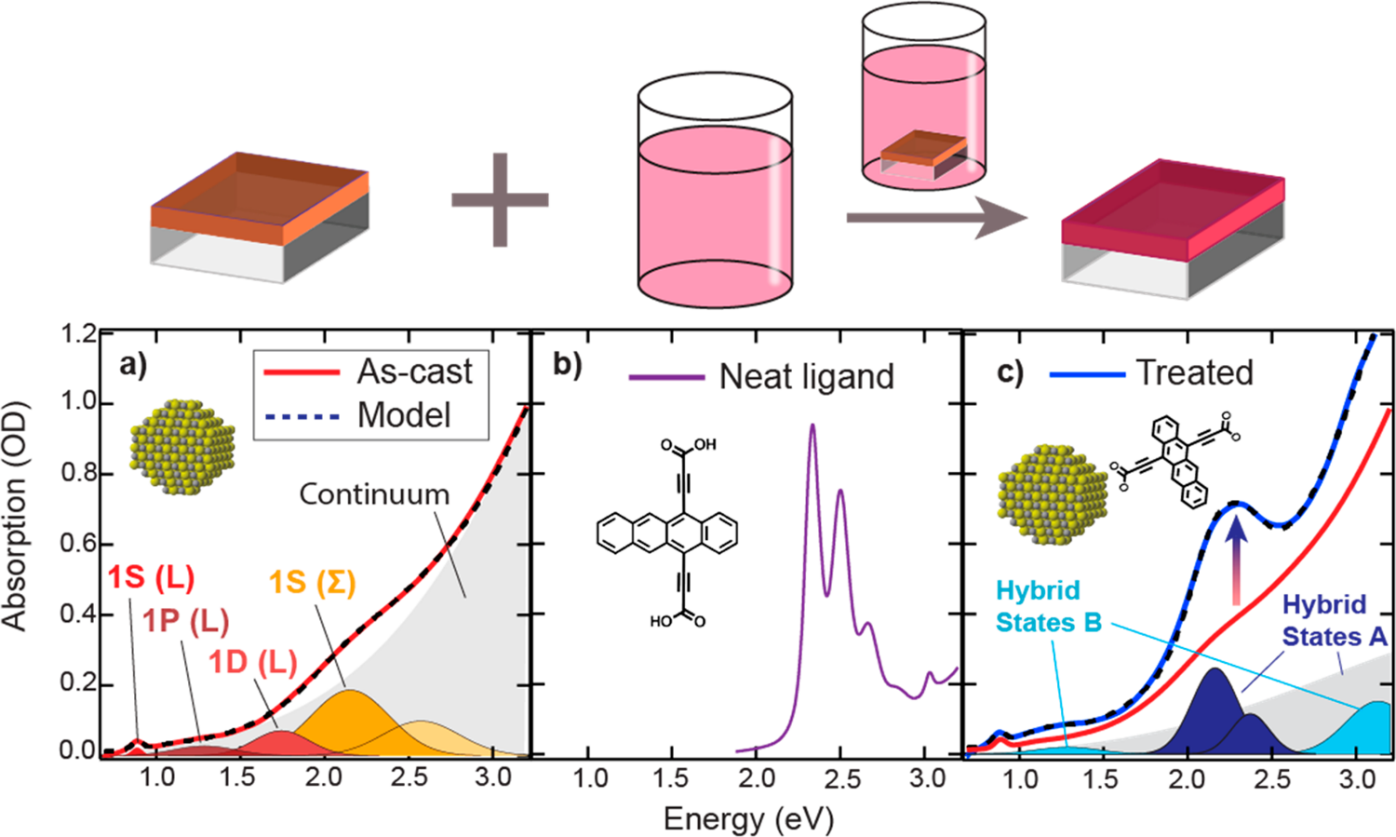
Absorption spectra and schematic of solid-state ligand exchange procedure. The features in the as-cast film spectrum (a) are modeled using the previously derived PbS QD electronic states (ref (32)) and are shown in red and yellow, as well as the continuum, shown in gray. (b) The chemical structure of Tc(Ac-COOH)_2_ and neat ligand spectrum (50 μmol/L) in DMF (c). The QD film spectrum (blue) after treatment in 0.125 mmol/L Tc(Ac-COOH)_2_ as well as the modeled spectrum (black dotted line) and as-cast spectrum (red line) for reference.

We find strong evidence for electronic state mixing between the QD and the discrete molecular states of the surface-bound moieties. The orbital hybridization manifests in two ways and depends on the ligand orientation with respect to the QD surface.^28−31^ First, we find, as in previous studies, a broad-band absorption enhancement due to the coupling of the discrete molecular states with the broad continuum of states in the QD. Second, in addition to the broad-band enhanced absorption, we find that tetracenedicarboxylic acids can mix with a narrow band of higher lying QD states, which we assign to the excitonic states that form from the Σ-valley conduction band, producing hybrid states with more narrow resonances, and the strength of that electronic coupling depends upon the orientation of the ligands relative to the QD surface. Both observations indicate strong electronic coupling and can be described within the Newns–Anderson–Grimley (NAG) model.^28^ We also performed density functional theory (DFT) simulations of the diacid ligand bound to PbS surfaces and found a strong preference for the ligand to lie flat on the PbS surface and interact electronically through the π-system of the tetracene ligands.

## Results

### Steady-State UV–Vis Absorption

In past studies we have employed spectrophotometric absorption titration experiments to follow and study ligand exchange reactions *in situ*.^22^ In those experiments, the incoming ligand induces a broad-band increase in the absorption of the QD/ligand solution. The strength of the enhanced broad-band absorption was found to quantitatively track the ligand exchange;^15^ thus, by tracking the enhanced absorption, the ligand exchange progress can also be followed and then ligand exchange isotherms constructed and analyzed.^23^ Here, a solid-state ligand exchange spectrophotometric experiment was used to track the ligand exchange reactions, and the process is depicted in Figure 1. A series of 9 films were prepared by spin-casting in a nitrogen-filled glovebox from a 75 mg/mL PbS QD stock solution (details can be found in the Supporting Information), and the absorption spectra of the 9 films were measured prior to treatment. Figure 1a shows an example of one as-cast (pretreatment) film. The spectra of the as-cast films display a QD exciton at 0.90 eV, corresponding to a 5.0 nm diameter particle,^32^ and the spectra are modeled by a series of five Gaussian peaks: three (Figure 1a, red-shaded deconvolved peaks) represent excitons derived from the L-valley conduction and valence bands, i.e., the 1S(L), 1P(L), and 1D(L) excitons, and two (Figure 1a, orange-shaded deconvolved peaks) represent excitons derived from the Σ-valley, i.e., the 1S(Σ) and 1P(Σ) excitons. A broad background (Figure 1a, gray-shaded region) was needed to reproduce the absorption spectrum, which is assigned to the continuum within both the L- and Σ-valleys. In our model of the as-cast spectrum (Figure 1a, black-dashed trace), the energy positions of each Gaussian were fixed based upon a **k·p** calculation of excitons in both the L- and Σ-valleys and is described elsewhere,^33,34^ and a similar modeling of PbS QD spectral features based on the electronic states was shown by Kennehan et al.^35^ The spectrum of neat Tc(Ac-COOH)_2_ (4 μmol/L) shows four sharp transitions arising from the S_0_–S_1_ vibronic manifold (Figure 1b). The as-cast film was soaked in a solution of Tc(Ac-COOH)_2_ in dimethylformamide (DMF) (for the experiment depicted in Figure 1, 0.125 mmol/L was used). The films were rinsed in neat DMF after soaking, thereby removing any unbound ligand from the resulting films. Removal of the unbound ligands is a critical step, because it isolates the spectral signatures to exclusively bound moieties of the QD/ligand complex. After washing, the absorption spectrum was collected for the exchanged film (Figure 1c, blue trace).

The UV–vis spectra of the treated films, in general, show a broad-band increase in optical density as well as several new features. The dominant feature is a new absorption peak at around 2.2 eV, attributed to the surface-bound ligand species. In comparison to the neat ligand spectrum (Figure 1b), the Tc(Ac-COOH)_2_ absorption line shape is significantly broadened and red-shifted (Figure 1c, dark blue shaded peaks). The observed broadening of the molecular vibronic features is an indication of the strong electronic coupling between the semiconductor and the molecular electronic states of the surface-bound species.^36−41^ We found that this broadening of the vibronic line shape is distinct to the diacid character of the attached ligands by preparing QD films treated with TIPS-Tc-COOH (i.e., the monoacid version from ref (12)), which results in significantly narrower vibronic peaks (Figure S1). Modeling of the post-treatment spectrum (Figure 1c, black-dashed trace) was done by starting with the model of the as-cast film (Figure 1a, black-dashed trace and Figure 1c, red trace) described above and adding four Gaussians that are distinct to the postexchanged spectrum. In addition, the broad-band continuum absorption described above was allowed to increase to capture the broad-band absorption enhancement (Figure 1c; the broad gray shaded peak represents the broad-band absorption enhancement). The additional peaks observed in the treated spectrum (Figure 1c), as determined by the model, are a doublet centered at 2.2 eV (Figure 1c, dark blue shaded peaks) and one peak on either side of the 2.2 eV band at ≈1.5 eV and ≈3 eV (Figure 1c, light blue shaded peaks). We find that the central 2.2 eV peaks representing the ligand singlet transition overlap the PbS Σ-valley 1S exciton transition at 2.2 eV (1S(Σ) in Figure 1a). The two satellite peaks (1.5 and 3.0 eV) are separated equally from the central peaks by approximately 700–800 meV. We postulate that the broadening of the bound ligand absorption and the newly formed satellite peaks that are in neither the neat ligand nor the as-cast spectrum are a result of strong electronic hybridization between the tetracene and PbS QDs.

**Figure 2:**
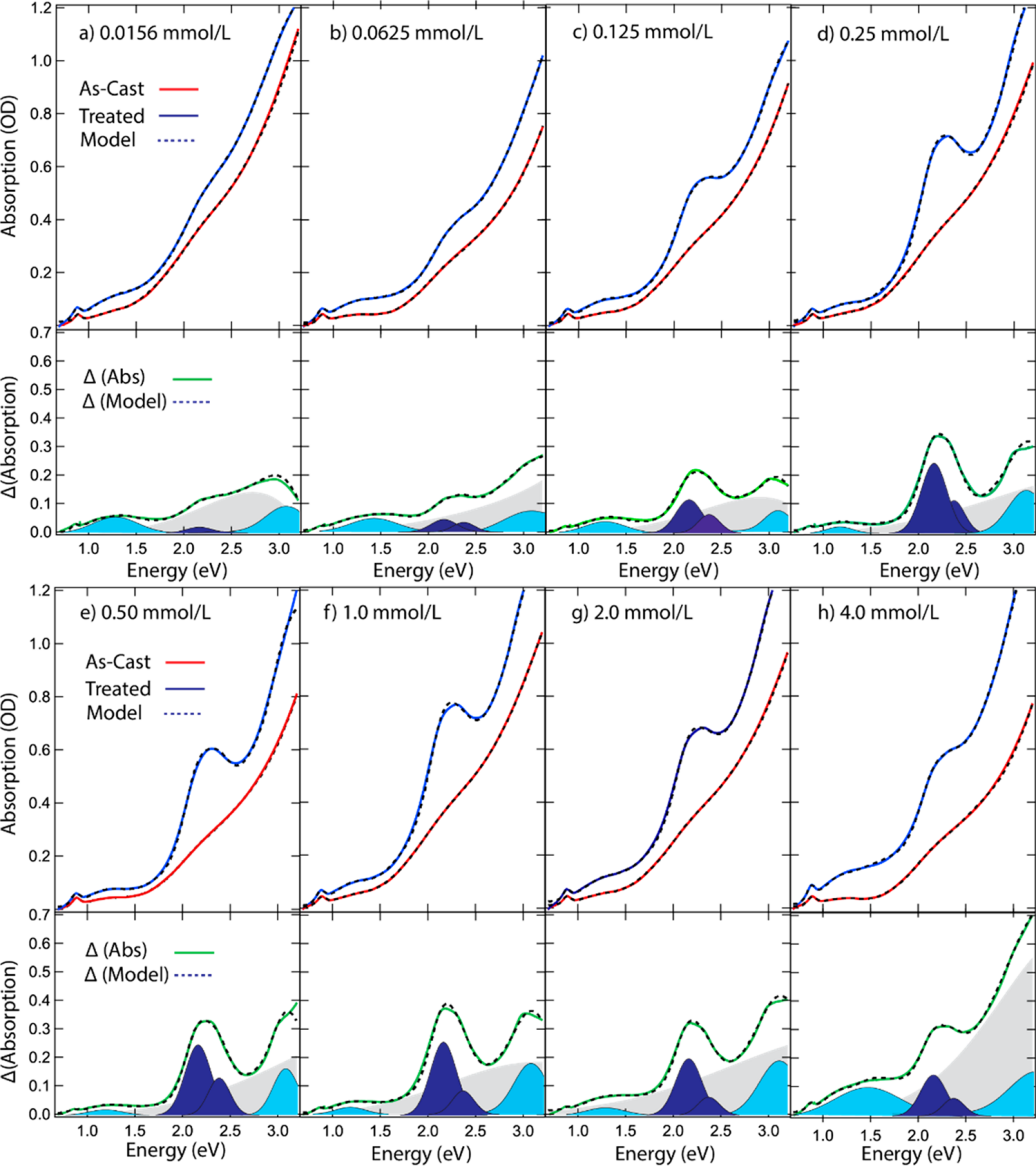
(a–h) Top panels: experimental (solid traces) and modeled (dotted traces) UV/VIS/NIR absorption spectra as optical density (OD) of the pretreated (red) and post-treated (blue) ligand exchanged films at each concentration. (a–h) Bottom panels: Δ(Abs) spectra produced from the difference of the post- and pretreated films (green trace), reproduced only from the peaks that arise from Tc(Ac-COOH)_2_ treatment and absorption enhancement (dotted line), and excluding those from the PbS core.

The procedure described above was then repeated for a variety of input ligand concentrations. To do this, a series of eight as-cast QD films was prepared from a stock solution. The initial absorption spectrum of each film was recorded prior to exposing the film (Figure 2a–h, red traces) to the ligand exchange solutions of various ligand concentrations. We observe small differences in the as-cast absorption spectra from film to film. The differences are captured in the shape of the feature assigned to the continuum absorption (gray shaded region in Figure 1a), which we ascribed to differences in variations in QD size and quality and/or differences in inter-QD coupling. The excitonic features, energetic positions, and widths of the 1S(L), 1P(L), 1D(L), 1S(Σ) and 1P(Σ), described for Figure 1, are held constant in our modeling (Figure 2a–h, dashed traces) for each film. Each film was individually soaked in a Tc(Ac-COOH)_2_ solution in DMF with varying concentrations of the ligand, from 0.0156 to 4 mmol/L (Figure 2a–h, blue traces). Figure 2a–h (top panels) displays the resulting absorption spectra of the QD films before (red) and after treatment (blue) in the diacid solutions, and each was modeled using the same procedure as described for the spectrum in Figure 1c. Additionally, we show the Δ(Abs) spectrum in Figure 2 (bottom panels), produced by subtracting the pretreated spectrum from the post-treated spectrum, and display the four absorption features induced by the ligand exchange and discussed above. The four peaks plus an increase in the broad-band contribution reproduce the Δ(Abs) (black-dotted traces). Here the positions and widths of the Gaussian are held constant for each of the films, and only the intensity is allowed to vary. The Δ(Abs) and peak analysis reveal the concentration dependence of the relative intensities of the central 2.2 eV transitions as well as the satellite peaks (presented and discussed below in Figure 5a). The ligand absorption intensity clearly varies nonmonotonically with solution concentration, as do the satellite peaks. From low to intermediate concentration (0.015–0.5 mmol/L) the spectrum changes from being dominated by the satellite peaks to central peaks dominating. Then from 0.5 to 4 mmol/L, the trend flips; the central peak decreases as the satellite peaks become the dominating species once again. We have labeled the central peaks as arising from hybrid state A and the satellite peaks as arising from hybrid state B in Figure 1c. Thus, instead of having a monotonic response of the 2.2 eV bound tetracene absorption feature (hybrid state A) with the ligand-exchange solution concentration, we observe at least two phases of ligand exchange with distinct optical and electronic properties: intermediate (0.125–0.5 mmol/L) and high (1–4 mmol/L) concentrations, with the third possible phase at low concentration (<0.125 mmol/L). In the intermediate concentration phase, addition of Tc(Ac-COOH)_2_ increases the relative proportion of A compared to the B peaks, and at the high-concentration phase, addition of Tc(Ac-COOH)_2_ increases the relative dominance of B relative to A, as well as the broad-band absorption enhancement. Note that the scattering component, discussed above, may also change upon ligand treatment and these small changes are captured in the enhanced broad-band absorption. Finally, the QD exciton transition energy as a function of Tc(Ac-COOH)_2_ was determined (Figure S2) and does not follow a strong trend. To gain further insight into the ligand binding geometries, we measured the FTIR absorption for each of the films and employed DFT modeling of various binding geometries.

### Fourier Transform Infrared (FTIR) Spectroscopy and DFT Simulations

**Figure 3:**
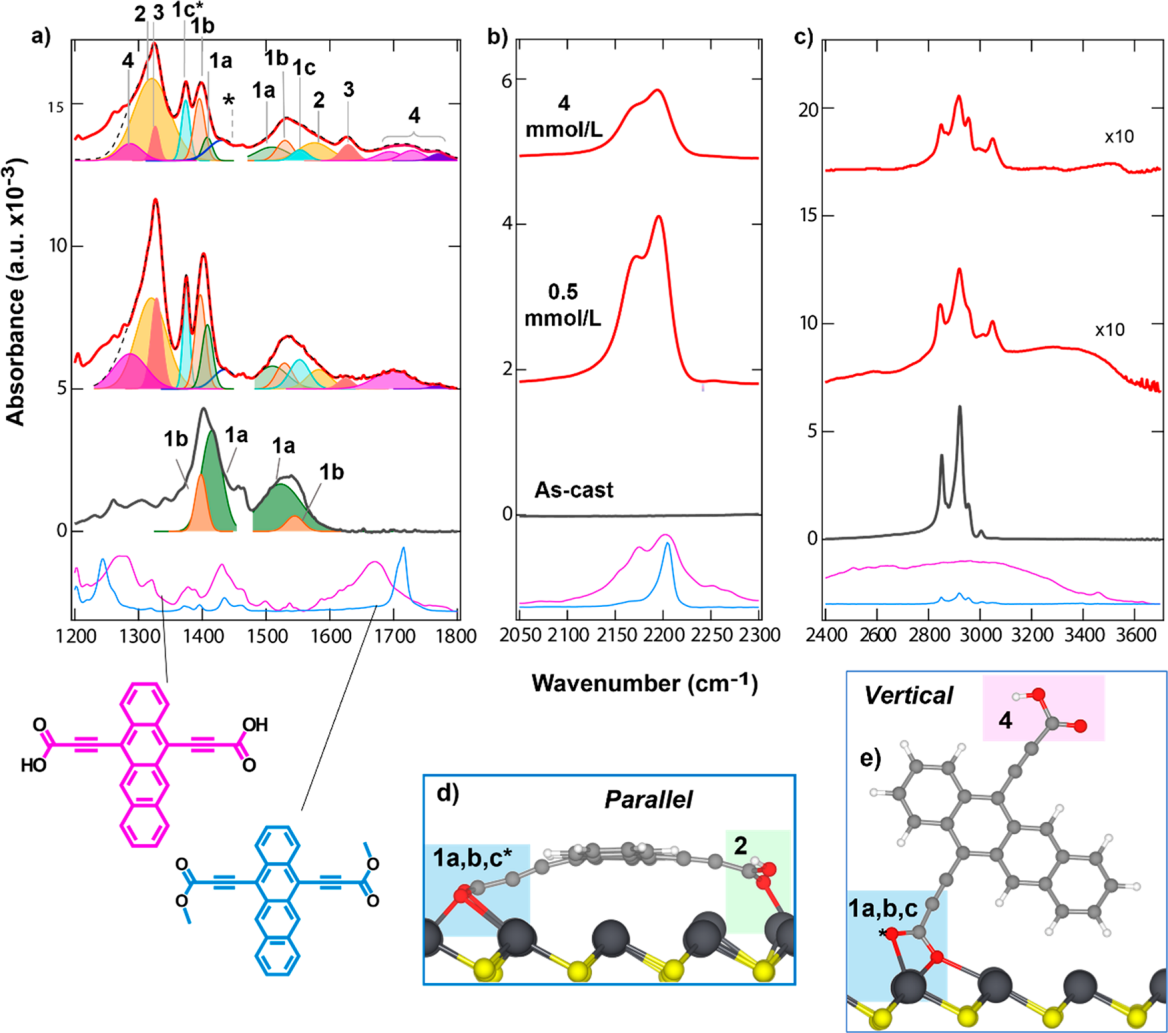
Experimental (solid) and modeled (dashed) FTIR spectra of the (a) carboxylate, (b) alkyne, and (c) aliphatic and hydroxide regions of Tc(Ac-COOH)_2_ (pink) and Tc(Ac-COOMe)_2_ (light blue) solids, as-cast, and 0.5 and 4 mmol/L exchanged films (from bottom to top). (a) includes the peaks determined in the model with the labels for the assignments as shown in the colored squares in (d) and (e). The neat ligand spectrum (pink) was acquired on a powder sample in ATR mode, and the methyl ester derivative (blue) sample was a drop-cast film in transmission mode. The optimized parallel (d) and vertical (e) binding geometries are shown with the COO modes labeled.

FTIR characterization provides semiquantitative information about the amount, identity, and binding geometry of the ligands on the QD surface. The experimental transmission FTIR spectra (Figure 3a–c and Figures S8–S11) were acquired on the same films as those from the absorption experiments and include the Tc(Ac-COOH)_2_ powder (DRIFTS-FTIR) spectrum and the drop-cast methyl ester derivative [Tc(Ac-COOMe)_2_] of the tetracenedicarboxylic acid. Spectra of lead oleate, oleic acid, DMF, and the complete concentration series can be found in Figures S8–S11. For the data analysis of the exchanged films, we only discuss here the peaks that change upon ligand exchange; a complete list of peak assignments is given in Table S2. DFT calculations, using periodic slab models of PbS surfaces, were employed to probe the adsorption geometries of Tc(Ac-COOH)_2_ on PbS(111) and PbS(100) (see the Supporting Information for calculation details). The simulated bound Tc(Ac-COOH)_2_ molecules were anchored to the PbS surfaces through a deprotonated COOH group (see Figure S4 for adsorption energies and geometries of different binding modes). The results indicate that tetracene preferentially adsorbs at hollow sites on the (111) facet, where the deprotonated carboxylate moiety interacts with multiple Pb atoms through a combination of bridging and chelating modes. In contrast, adsorption on the (100) facet occurs on top of Pb atoms in a bidentate bridging configuration.

#### Carboxylate Region

**Figure 4:**
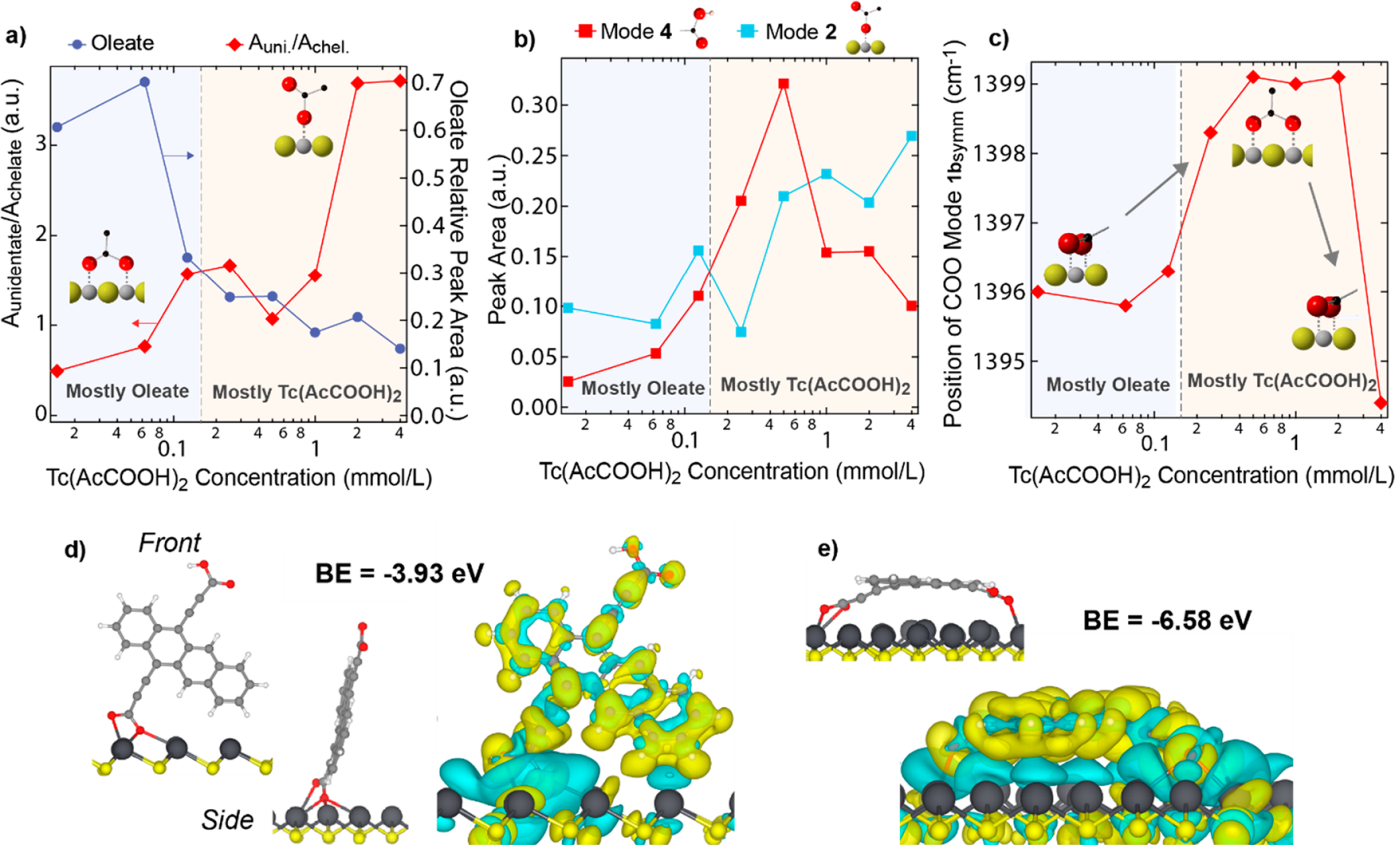
Diacid concentration dependence of (a) the ratio of the unidentate **3** to chelating **1a** mode symmetric peak areas (red diamonds) and the loss of CH intensity associated with the oleate ligand (blue circles). (b) Protonated (red) and unidentate (blue) mode peak areas and (c) frequency of the **1a** mode across the concentration series. The dashed vertical lines in (a–c) indicate where the ligand shell converts from majority oleate to majority Tc(Ac-COOH)_2_. Front and side view images and binding energies (BE) of (d) vertical and (e) parallel adsorption modes of deprotonated tetracene on PbS(111), with accompanying charge density difference plots. Yellow indicates an increase in charge density, and blue indicates a depletion. Gray, white, red, black, and yellow spheres represent C, H, O, Pb, and S atoms, respectively.

Figure 3a shows the carboxylate stretching region of the as-cast (black trace) and 0.5 and 4 mmol/L QD films (red traces), where the symmetric (asymmetric) COO^–^ stretching mode is from 1200 to 1450 cm^–1^ (1450 to 1700 cm^–1^). The asymmetric–symmetric COO^–^ peak separation (Δν_COO_) indicates the nature of the carboxylate-to-metal bonding motif, where the general rules are that Δν_COO_ > 300 cm^–1^ is regarded as a unidentate motif, 100 cm^–1^ < Δν_COO_ < 300 cm^–1^ is a bridging motif, and Δν_COO_ < 100 cm^–1^ is considered a chelating motif.^42,43^ Additionally, the C–O and C=O frequencies present in the Tc(Ac-COOH)_2_ powder and methyl ester derivative support the peak assignment of the QD-bound ligand vibrations. The as-cast, oleate-terminated QDs have two dominant COO^–^ stretching peaks. The asymmetric COO^–^ peaks at ν_asym_ = 1524 and 1547 cm^–1^ and symmetric COO^–^ peaks at ν_sym_ = 1415 and 1398 cm^–1^ are assigned to a chelating mode with Δν_COO_ = 109 cm^–1^ and a bridging mode with Δν_COO_ = 149 cm^–1^. These two COO^–^ modes associated with PbS/OA QDs have been observed previously and are labeled here as **1a** and **1b** (Figure 3a).^42,44^ There are no apparent unidentate or acidic oleate species present in the as-cast film. Treatment with the Tc(Ac-COOH)_2_ solution produces two main changes: (1) significant loss of intensity of the chelating, **1a**, mode and (2) the appearance of three additional binding modes, labeled **1c**, **2**, and **3**, as well as the appearance of free COOH modes, labeled as **4**. The additional binding modes are assigned to bridging (**1c**, Δν_*COO*_ = 173 cm^–1^) and unidentate modes (Δν_*COO*_ = 262 and 300 cm^–1^, **2** and **3**, respectively), and the free acid C–O–H mode is at ν_C–O_ = 1286 cm^–1^ (**4**). **1c** and the unbound acid modes (**4**) are relatively most intense in the 0.5 mmol/L film; then they decrease as the diacid concentration increases. The modes that continuously increase in strength throughout the full ligand concentration series are **2** and **3** and correspond to an increase in the unidentate mode as the Tc(Ac-COOH)_2_ replaces oleate (Figure 4b, blue squares and line). In our DFT simulations, we find that as the angle between the molecule and surface decreases to 0 (compare Figure 3d,e), the overall IR absorption intensity diminishes, and a unidentate binding species with Δν_COO_ = 262 cm^–1^ appears in addition to a bridging mode with Δν_COO_ = 150 cm^–1^. Thus, we associate the presence of binding mode **2** in our experimental results with a tilted or parallel Tc(Ac-COOH)_2_ configuration. In agreement, researchers have similarly found a unidentate geometry associated with a strongly tilted or parallel geometry of polycyclic dicarboxylate molecules on metal oxide surfaces.^45^ Under the assumption that the surface-coordinated carboxylate corresponds to the bridging and unidentate COO^–^ modes (**1**–**3**), the carboxylic acid corresponds to mode **4** and that the relative oscillator strengths are the same for all ligand orientations, an analysis of the relative peak areas can indicate the composition of bound versus unbound carboxylate. Compared to the starting amount of COO^–^ in the as-cast film, there is significantly more bound COO^–^ at intermediate concentrations (0.5 mmol/L), and roughly the same bound COO^–^ at high concentrations (4 mmol/L). These results are summarized in Figure S12c,d.^46^

#### Alkyne Region

Figure 3b shows the alkyne region of the three representative treated QD films, the neat Tc(Ac-COOH)_2_ powder, and the methyl ester derivative. The C≡C doublet frequencies are 2177 and 2203 cm^–1^ (Figure 3b, bottom panel). After binding to the surface, the alkyne peak at 2203 cm^–1^ red-shifts by 5.6 cm^–1^ while the 2177 cm^–1^ peak red-shifts by 9.5 cm^–1^. The alkyne stretching intensity maximum occurs at 0.5 mmol/L, and then the intensity decreases at concentrations >0.5 mmol/L. The decrease in intensity with increasing diacid concentration is consistent with the diacid tilting toward the QD surface at higher concentration. This decreased intensity for the parallel configuration is again captured in our PbS DFT simulations. A physical picture for the decreased oscillator strength is that in the vertical configuration the transition dipole is perpendicular to the QD surface and the surface image dipole is additive (vertical head-to-tail arrangement), while in the parallel configuration the image transition dipole is opposite (horizontal head-to-tail arrangement) and thus experiences a reduced intensity.^42,47^ We also confirmed this behavior in a separate experiment performed by measuring the FTIR spectra of a single film treated in one solution of 0.5 mmol/L ligand concentration and measuring the spectra at several time intervals during soaking in the ligand solution (Figure S13). In that experiment, the loading of diacid increases with increasing soaking time, indicated by the loss of the ethylene and methylene stretching intensity at 3000 cm^–1^ and increase of the alkyne peak. Identical to what we observe by soaking in the 4 mmol/L film (Figure 3), the intensities of the COO^–^ and C≡C peaks decrease after the last soaking interval, indicating a loading-dependent change in the diacid geometry.

#### CH and OH Region

Figure 4 shows the CH and OH stretching region. The prominent signature for oleate is the CH stretching band at 2750–3000 cm^–1^, the intensity of which is directly correlated to the amount of oleate bound to the QD surface since the diacid does not have methylene or ethylene groups. Treatment of the films in 0.0156 mmol/L Tc(Ac-COOH)_2_ results in a significant loss of the CH stretching intensity, which continues to decrease with increasing diacid concentration, confirming the effective removal of oleate upon binding of the tetracene ligand. In addition to the narrow peaks associated with CH stretching, we also observe OH stretching intensity and line shape that is concentration dependent. The OH features only appear at ligand concentrations >0.125 mmol/L and are either extremely broadened (0.25 and 0.5 mmol/L) or narrow (1–4 mmol/L). Since we do not observe an OH peak in the as-cast films, this strongly suggests that the native oleates are bound through the COO^–^ to the polar (111) facets, rather than the neutral (100) facets.^48−50^ By the same argument, the OH present in the diacid-treated films likely arises from isolated and surface-bound tetracene (uncoupled to a proximal Tc(Ac-COOH)_2_) that is protonated and unbound on one side of the molecule and anchored on the other. The extreme broadening of the OH stretch is a signature of hydrogen bonding, indicating that for these concentrations a significant population of the unbound carboxylic acid of the diacid participates in H-bonding with adjacent bound Tc(Ac-COOH)_2_^51−54^ in the intermediate-concentration regime. This signature decays at higher concentration as the monodentate configuration increases, implying that as the ligands tilt into the parallel configuration, the hydrogen bonding decreases.

#### Summary of FTIR and DFT Simulations

The FTIR data (summarized in Figure 4a–c) provide a picture of how the ligand binding geometry evolves as the Tc(Ac-COOH)_2_ replaces the oleate ligands. The sum of the CH modes (Figure 4a, blue dots and line) is indicative of the loss of oleate for increasing the diacid concentration. The lowest concentration range is characterized by an ≈40% loss of the oleate ligands with only a small addition of Tc(Ac-COOH)_2_, which we assign to partial removal of oleate by DMF. We observe >90% removal of oleate by 0.5 mmol/L, and at 4 mmol/L the oleate is nearly completely removed. Thus, DMF clearly assists in the ligand exchange; first DMF removes some oleate that is then only replaced by Tc(Ac-COOH)_2_ at higher concentrations. At the highest concentrations the remaining surface-bound oleate is removed by direct ligand exchange with the diacid.

Although a mixture of bridging and unidentate binding geometries exist at all treatment concentrations, the trend (Figure 4a, red circles and line) indicates that the unidentate/bridging geometry increases with increasing concentration, signifying, as discussed above, that there are more parallel diacids at the higher diacid concentrations. Next, we find that while the unidentate mode (**2**) continuously rises (Figure 4b, blue squares and line), the carbonyl corresponding to the protonated acid (**4**) has a nonmonotonic dependence, displaying a maximum at 0.5 mmol/L (Figure 4b, red squares and line), after which it decreases. We ascribe this decrease to a more parallel configuration; i.e., when the ligands are perpendicular to the PbS surface they can hydrogen bond, and when they are tilted parallel to the PbS surface they cannot, consistent with both the trends in OH stretching, i.e., less hydrogen bonding in the parallel configuration, as well as the alkyne region, i.e., lower intensity that is more red-shifted. Additional evidence of concentration-dependent tilting of the diacid on the QD surface is shown by the frequency of the chelating mode **1b** (Figure 4c). The upright chelating binding motif corresponds to a greater bond strength of the (COO^–^)–Pb bond, whereas this chelating bond is weakened by the tilting of the molecule at the surface. This change in bond strength is observed as a decrease in the chelating mode **1b** frequency, where the bond strength is greatest at intermediate concentrations (vertical geometry) and weakest at the low and highest concentrations (parallel geometry).

In our DFT simulations, as the angle between the aromatic ring system and the surface decreases from ≈90° (vertical) to 0° (parallel), the tetracene ligand binds more strongly to the PbS surfaces with the parallel adsorption energy being more favorable than the vertical mode by 2.65 eV on PbS(111) (see Figure 4d,e for charge density plots). The preference for parallel Tc(Ac-COOH)_2_ is attributed to the increased interaction between the aromatic rings and the PbS surface and the formation of an additional monodentate bond through the protonated carboxylate group. For all adsorption geometries, Tc(Ac-COOH)_2_ adsorbed more strongly to the (111) facet than to the (100) facet of PbS, likely due to the polarity of the PbS(111) surface; consistently, charge density difference plots indicate that the aromatic ring system interacts more strongly with the (111) facet than the (100) facet (see Figure S5).

### Structural Characterization of Ligand-Exchanged QD Films

**Figure 5:**
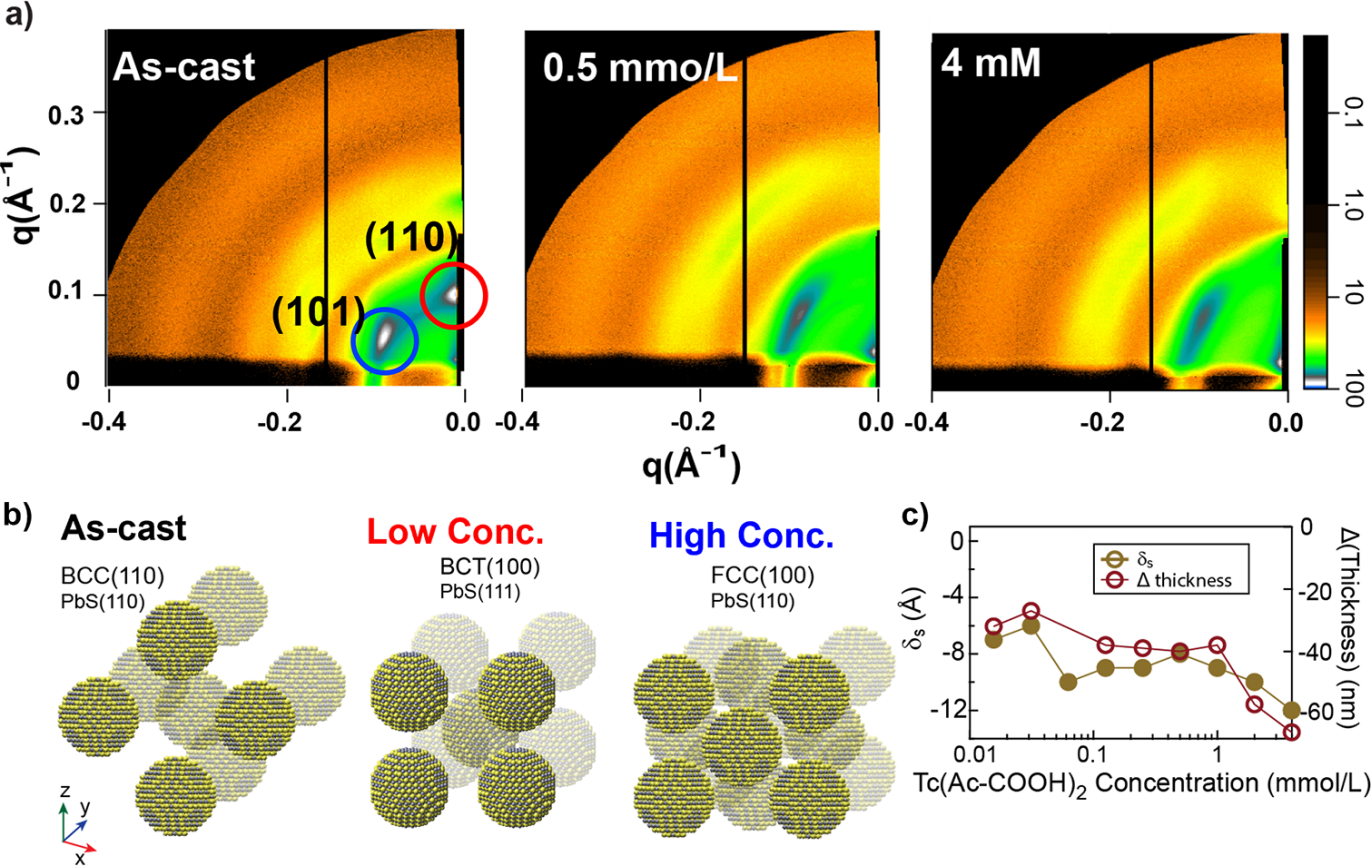
Structural characterization of the film. GISAXS (a) patterns of the low-, intermediate-, and high-concentration regions. The indices are labeled, and the red circles in the oleate-terminated film are the predicted scattering vectors for the BCC(110). Cartoon representation (b) of the change in the superlattice upon exchange at low concentrations. The QD spacing (gold, solid circles) and film thickness (red, open circles) are summarized in (c).

To study the physical properties of the films, ellipsometry, grazing-incidence small angle X-ray scattering (GISAXS), and wide-angle X-ray scattering (GIWAXS) measurements were performed on a set of samples prepared identically to those used for optical studies but were deposited on Si substrates for the GISAXS measurements. A QD film was soaked in neat DMF for 4 h as a control. From the GISAXS/WAXS data, we observed that both the QD superlattice and PbS crystal lattice orientation change upon the binding of Tc(Ac-COOH)_2_. Shown in Figure 5a are the GISAXS patterns of the as-cast films and exchanged films from the low-, mid-, and high-concentration regimes, as described from the absorption and FTIR results. The oleate-terminated PbS superlattice in the as-cast film features a pattern well described by a body-centered-cubic (BCC) superlattice, with the [110] direction along the surface normal, which is typical for particles of this size,^55−57^ and the PbS crystal lattice similarly has the [110] orientation along the surface normal, consistent with alignment of the ⟨111⟩ PbS crystal directors along the BCC near-neighbor directions (see WAXS in Figure S14).^58^ Exchange at all concentrations leads to an increasing film density as the superlattice rearranges from the BCC to a mixture of body-centered-tetragonal (BCT) and face-centered-cubic (FCC) with an increase in paracrystallinity. The shift in packing is reflected in the movement of the BCC (110) upward toward the (200) of the FCC or BCT. The disorder precludes a quantitative analysis of the mixture, but both FCC and BCT structures have a [100] orientation along the surface normal (see Figure S15). The higher coordination of the FCC superlattice typically results in orientation disorder of the contained nanocrystals. At low concentrations, the superlattice appears to favor BCT with compression of the *a* dimension along the surface normal, while at high concentrations, it is mostly FCC. A cartoon depicting the superlattice and atomic lattice rearrangement is shown in Figure 5b.

The QD spacing (δ_s_; surface-to-surface distance) in the films is determined from the [110] and [111] planes in the GISAXS patterns of the as-cast and exchanged films, respectively, and the thickness was determined using ellipsometry. We assume a particle diameter of ∼5.3 nm when computing the spacing from the superlattice spacing. The film thickness (Figure 5c, red open circles) and δ_s_ (Figure 5c, brown solid circles) follow the same trend. The as-cast film thickness is 186 ± 2 nm (175 ± 1 nm for the DMF control), and δ_s_ is 21 ± 0.2 Å, approximately the length of a single oleate ligand, indicating that the ligands are interdigitated in the film.^59^ After the films are treated in the low-concentration diacid solution, the thickness dramatically contracts by ≈20%, and the QD spacing, δ_s_, decreases to 14 ± 0.2 Å. At intermediate concentrations, both the film thickness and particle separation decrease, with δ_s_ being about 1 nm, followed by a further densification of the film slightly, to <1 nm. The final δ_s_ in the 4 mmol/L film is 9 ± 0.1 Å.

### Sub-picosecond Excited-State Dynamics

**Figure 6:**
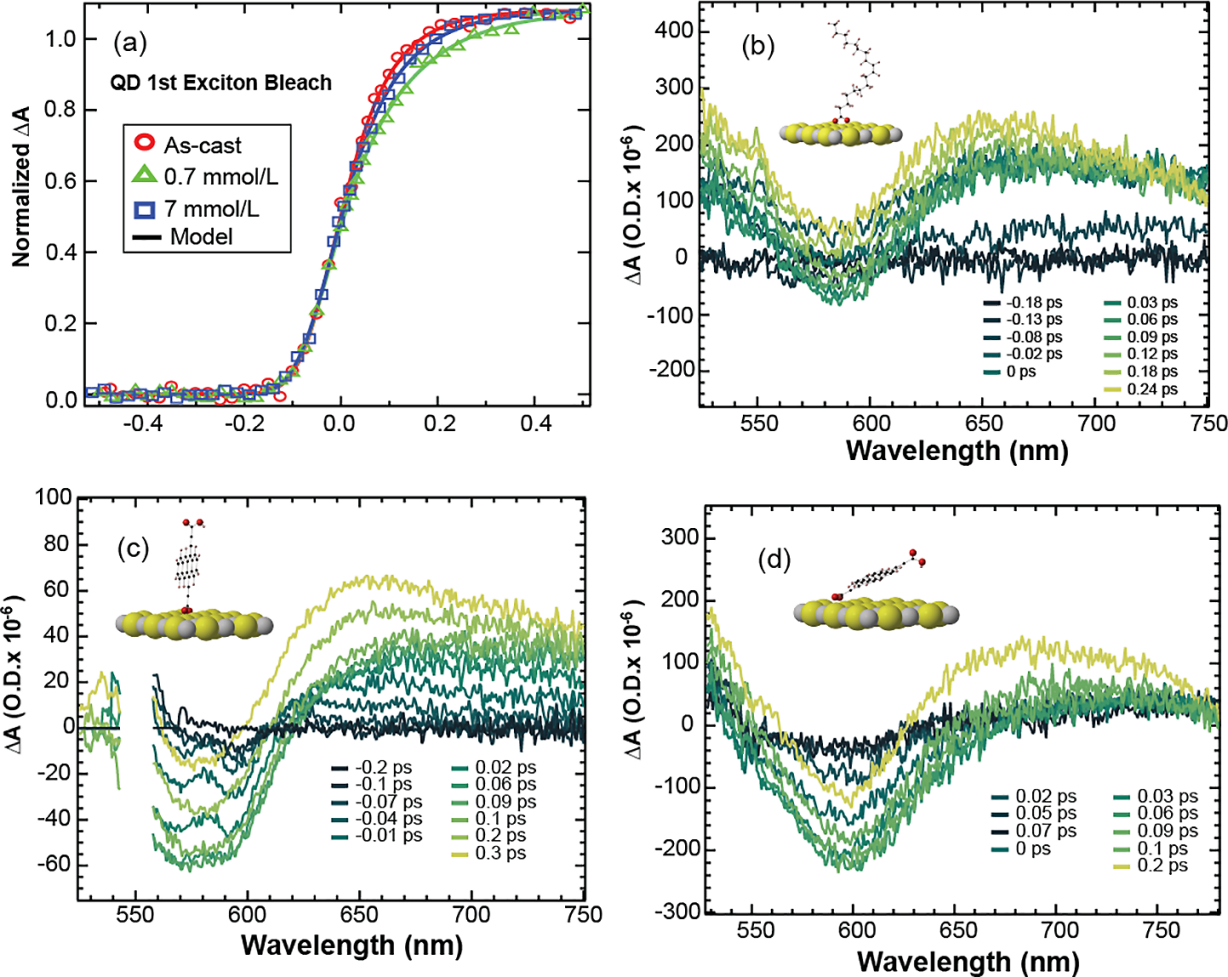
Ultrafast dynamics of the treated films detected in the NIR and visible regions under 550 nm (2.25 eV) excitation. (a) Normalized kinetic traces of the QD first exciton bleach for 0.7 mmol/L, 7 mmol/L, and as-cast films. Spectral slices of the (b) as-cast, (c) 0.7 mmol/L, and (d) 7 mmol/L films. Insets in parts (b–d) are cartoons of dominant population ligand geometry on a PbS monolayer at the given Tc(Ac-COOH)_2_ treatment concentration.

Shown in Figure 6 are the QD bleach dynamics recorded at 1550 nm and time-resolved spectra from transient absorption (TA) spectroscopy of an as-cast and 0.7 and 7 mmol/L films excited at 550 nm (2.25 eV). Based on the linear absorption spectra and FTIR analyses presented earlier, the 0.7 mmol/L film has a greater population of vertical Tc(Ac-COOH)_2_ species, whereas the 7 mmol/L film has more parallel and tilted species. Excitation at 550 nm can excite the Tc(Ac-COOH)_2_ singlet, the PbS Σ-band transition, or the hybridized transition, and the population of each species is determined by their relative absorption. We observe differences in the rise of the QD bleach among the three samples, but there is not a monotonic dependence, with the oleate/as-cast film showing the fastest rise, followed by the 7 mmol/L and the 0.7 mmol/L films having the slowest response. We modeled the rise of the bleach (smooth traces in Figure 6a) as an exponentially rising function convoluted with a Gaussian function that represents the instrument response function (IRF). All three films are modeled simultaneously, and the best-fit IRF is a Gaussian with a 115 fs full width at half-maximum (fwhm) and corresponds to the part of the data from −0.1 to 0 ps in Figure 6a. For the oleate-terminated as-cast film (Figure 6a, red circles), the bleach of the QD exciton transition reaches a maximum within 80 fs, which includes the arrival time into the lowest exciton state via carrier cooling from upper electronic states. The 0.7 mmol/L (Figure 6a, green triangles) and 7 mmol/L (Figure 6a, blue squares) films have rise times of 130 and 97 fs, respectively. In the visible TA spectrum of the as-cast film (Figure 6b), there are two main features: one very broad photoinduced absorption (PIA) that appears within about 100 fs and a bleach at 590 nm from the Σ-band, and both features have similar time scales. The 0.7 mmol/L film (Figure 6c) also has a bleach and photoinduced PIA, but the bleach is at 575 nm and exhibits a distinct spectrum with narrow features that form immediately upon excitation which we assume to be related to the vibronic structure of a molecular species. These molecular spectral features evolve into a broad bleach after about 300 fs. In contrast, the 7 mmol/L (Figure 6d) film has a broad bleach at about 600 nm that resembles that of the as-cast bleach. The 575–600 nm bleach in both treated films is much stronger compared to that of the as-cast film and is attributed to the presence of the Tc(Ac-COOH)_2_ ligand shell.

We interpret the difference of the as-cast and treated film dynamics at early delay times as being dependent on the nature of the ligand geometry with respect to the surface and the type and strength of ligand–QD coupling. The features of the molecular species present for about 200 fs in the 0.7 mmol/L film are indicative of a singlet excitation on a Tc(Ac-COOH)_2_ monomer in the vertical geometry that is not as well electronically coupled to the QD. Following the singlet generation on the monomer, conversion of the structured bleach to the broad feature could either be delocalization of the singlet to adjacent surface-bound Tc(Ac-COOH)_2_ or transfer to the QD via charge or energy transfer. Furthermore, we postulate that the time required to transfer the molecular species to the QD slows the growth of the first exciton bleach. On the other hand, in the 7 mmol/L film, where the dominant ligand geometry is parallel/tilted on the surface, the 600 nm bleach has no molecular features. Rather, the 7 mmol/L film spectra contain signatures of the coupled system, and exciting the ligand quickly delocalizes to the QD/ligand hybrid state, thereby also placing the bleach rise time between those of the as-cast and the 0.7 mmol/L films.

## Discussion

We previously found that carboxylate ligand exchanges proceed on PbS QD surfaces via an X-type ligand exchange where the incoming acid exchanges a proton with the outgoing acid in an essentially “one for one” fashion, with each oleate being replaced by an incoming carboxylate.^22^ In the present case, due to the diacid functionality, there are three scenarios that could satisfy the above criterion: (1) one Tc(Ac-COOH)_2_ replaces one OA, resulting in one bound COO^–^ and one free COOH, (2) one diacid replaces two oleates, and the surface exchange ligand lies parallel to the surface with two bound COO^–^, and/or (3) one diacid replaces two oleates on neighboring QDs and cross-links the two QDs, where the ligands are in the vertical geometry with two bound COO^–^ groups. Considering the steric situation, the native oleate density in the as-cast films is about 3 OA nm^–2^. Based on the dimensions of the tetracene diacid molecule and considering only steric effects, if all the diacids are in the completely parallel geometry, then it could achieve 2 Tc(Ac-COOH)_2_ binding sites per nm^2^ at best (see Figure S20 for dimensions of ligand on the PbS surface). Thus, a completely parallel configuration where all Tc(Ac-COOH)_2_ lie flat is not likely. In our DFT simulations, we find a much stronger binding energy for the parallel geometry compared to the vertical geometry, indicating a significant increase in interaction of the ligand with the QD in the parallel geometry (Figure 4d,e). However, we note that our DFT simulations represent an idealized situation of a single Tc(Ac-COOH)_2_ bonding to one PbS surface. We do not account for nearby ligands bound to the surface that could interact through ligand–ligand interactions, nor do we account for solvent molecules or another nearby QD. Alternative to a completely parallel configuration, a tilted geometry (where the angle between the aromatic system and the surface is between 0 and 90°) is also possible. DFT simulations of the tilted geometry find a binding energy between those of the vertical and parallel geometries. These upright/tilted modes have smaller steric requirements than the parallel mode; furthermore, their ability to cross-link QD surfaces by adsorbing through both COOH anchoring groups could result in binding energetics comparable to the parallel mode.

**Figure 7:**
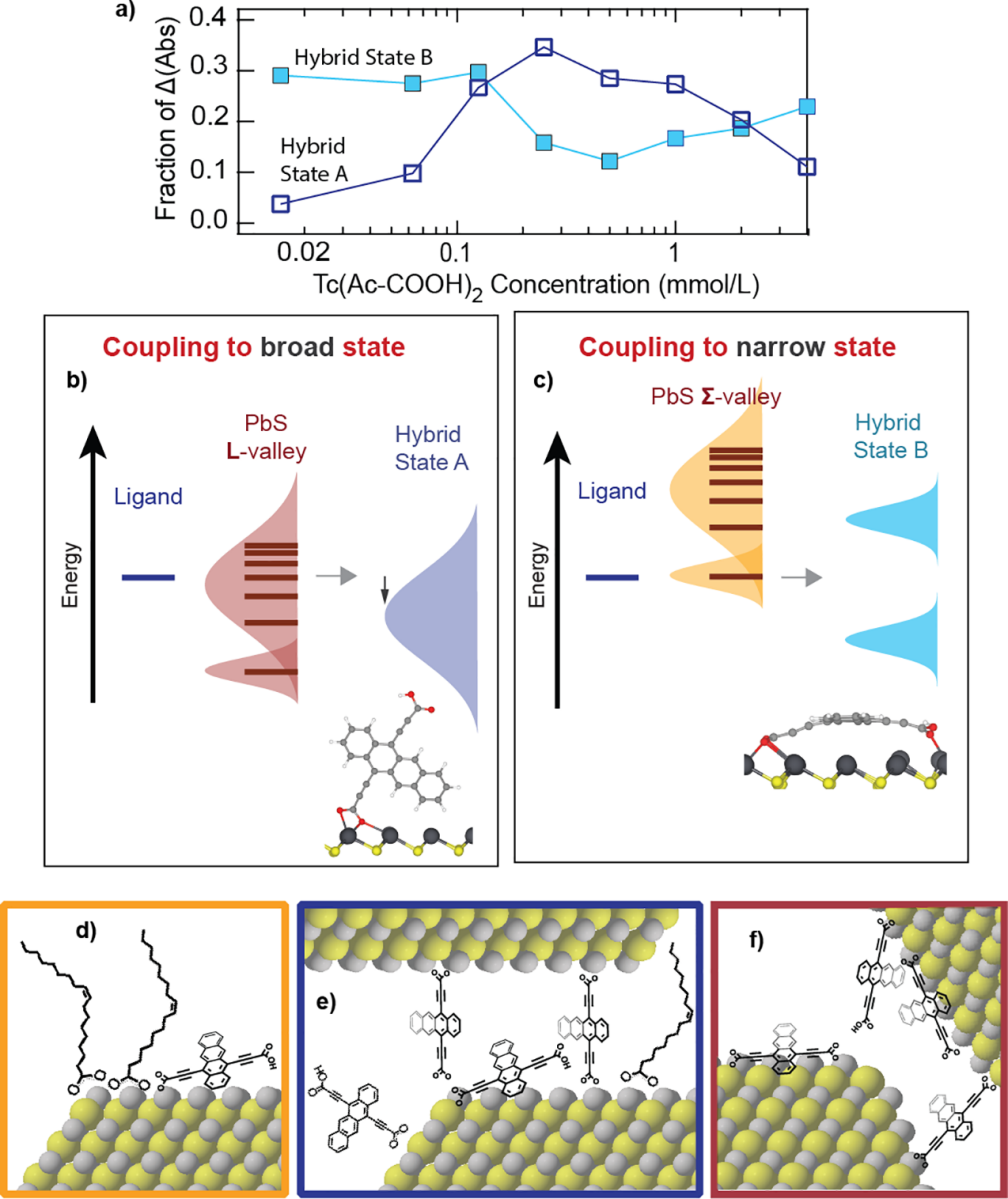
(a) Concentration-dependent change in linear absorption arising from PbS/Tc(Ac-COOH)_2_ hybrid states, and a depiction of the PbS/Tc(Ac-COOH)_2_ electronic coupling scenario of (b) hybrid state A and (c) hybrid state B, presented in the form of the Newns–Anderson–Grimley model. The structures of surface adsorbed Tc(Ac-COOH)_2_ shown were generated from the DFT optimized geometries from Figure 3. (d–f) Cartoon representations of the ligand–QD interface at (d) low, (e) mid, and (f) high Tc(Ac-COOH)_2_ regimes.

To search for support for the specific binding modes, we consider the experimental evidence based on FTIR, GISAXS, and TA. We observe stark differences in sample characteristics vs ligand concentration and define three regimes: a low concentration, an intermediate concentration, and a high concentration. At the lowest ligand concentrations, the ligand orientation is more difficult to determine experimentally due to the lower density of ligands, but there is clear evidence in the FTIR data of the ligand tilting, and it is likely that steric effects are less important in this regime, and therefore the ligands are able to find their lowest energy configuration. Meaning, removal of a small percentage of oleate by DMF and low coverage of Tc(Ac-COOH)_2_ both reduce steric effects of the parallel binding geometry. Further, since the oleate ligands determine the QD spacing at these lower concentrations, cross-linking is less probable; thus, a parallel geometry is more likely. At intermediate concentrations where the ligand shell is primarily Tc(Ac-COOH)_2_, we find that there is a mixture of upright and parallel Tc(Ac-COOH)_2_ structures, with the potential for intraligand cross-linking by forming surface-bound H-bonded dimers with neighboring ligands. Photoexcitation at this stage produces a distinct monomer singlet species that slows the rise of the QD bleach. For the high-concentration-treated films that are fully exchanged with Tc(Ac-COOH)_2_, there is a significantly less acidic COOH, fewer H-bonded groups, weaker alkyne IR stretching intensity, and the QD spacing decreases further, implying that there is a larger fraction of tilted or parallel arranged ligands. Photoexcitation at the high-concentration stage directly excites a hybrid state with mixed features of ligand and quantum dot, and the QD bleach rise kinetics are between those of the as-cast and 0.7 mmol/L films. Thus, at both the highest and lowest ligand concentrations there are more tilted, parallel oriented ligands while at intermediate ligand concentrations there are more up-right vertical oriented ligands. This apparent nonmonotonic behavior of the ligand configuration with input ligand concentration is likely a result of the various factors that determine ligand binding and geometry, including steric effects, mixture of oleate and diacid, and energetic effects, as well as the solution dynamics of the incoming ligands. Crude cartoon depictions of the dominant ligand structures in the low-, mid-, and high-concentration regimes are shown in Figure 7d–f.

We can return to the concentration-dependent UV/vis/NIR absorption spectral features (Figure 2) and explain the nonmonotonic behavior as a result of different ligand configurations. The concentration determines the relative amount of the “central” (labeled A) to “satellite” (labeled B) peaks, and in Figure 7a we display the relative contribution to the Δ(Abs) of two types of hybrid states, determined from the analysis of Figure 2. At low concentrations, we find a larger fraction of B states (Figure 7a filled squares) where A (Figure 7a open squares) is low. In the intermediate concentration range, A is high and B is low, and then at higher concentrations, B is higher than A again. Based on our analysis of the ligand binding geometry, we now associate A with the vertical geometry and B with the parallel geometry. Note that A is always present, indicative of the notion discussed above that there is always a mixture of vertical and tilted/parallel oriented ligands, but the contributions of A and B are anticorrelated. These drastic spectral differences in the parallel and vertical configurations can give insight into the mechanisms of ligand/QD hybridization and explain the A and B type responses. The A feature corresponds to the molecular states that are slightly broadened and red-shifted by the electronic interaction with the QD, similar to what was shown in ref (60), while for B the orbital hybridization is strong enough to observe splitting of the molecular states for those ligands in the parallel geometry. From our absorption modeling, the two satellite peaks equally split from the molecular state by ≈700 meV, indicative of strong coupling. Such a strong coupling of ≈900 meV has also been observed for molecules absorbed onto metal oxide surfaces, resulting in drastic changes to the absorption spectrum similar to what is observed here.^61^ The large splitting results from the increased electronic interaction of the ligand when in a parallel configuration; this interaction can be visualized from our DFT simulations. Figure 4b,c shows the ground state electron density difference, calculated from the difference between electron densities of the isolated Tc(Ac-COOH)_2_ and PbS slab. The Δ(charge) density map of the vertical configuration shows the carboxylate-alkyne arm of the ligand as the primary moiety involved in bonding, whereas the Δ(charge) density of the parallel geometry shows the entire tetracene backbone involved in bonding with the PbS surface. As the angle between the ligand and surface increases, the bonding interaction becomes localized on the carboxylate-alkyne arm of the Tc(Ac-COOH)_2_. Thus, in the parallel geometry there is a large interaction with the π-system of the tetracene backbone.

To describe both of these types of coupling in a tight binding approach (i.e., linear combination of molecular orbitals), we invoke the Newns–Anderson–Grimley (NAG) model,^28−31^ which describes interactions of molecular (or atomic) absorbates on metal and semiconductor surfaces. There are two limits of electronic coupling considered in the NAG model: (1) when the molecular states couple to a broad continuum of states in the substrate and (2) when the molecular states couple to a narrow distribution of states in the substrate. We noted previously that the molecular resonance has about the same energy as the 1S(Σ) of the PbS, and we hypothesize that it is coupling to these states that gives rise to the strong coupling. Figure 7b,c depicts the relative positions of the frontier orbitals of the Tc(Ac-COOH)_2_ (see the Supporting Information for methods) and both PbS 1S L and Σ states,^34^ respectively. We hypothesize that there are several possible electronic coupling pathways between the PbS and Tc(Ac-COOH)_2_, two that depend on the alignment of electronic states between the ligand and QD, and a third that is satisfied by the energetic resonance of the PbS Σ and Tc(Ac-COOH)_2_ singlet transitions. In the first two cases, the molecular state, which has a narrow density of states (DOS), couples to PbS electronic states that have either a narrow or broad DOS. Molecular states that couple to PbS states with a narrow DOS will produce hybridized states with split antibonding and bonding orbitals, and molecular states that couple to PbS states with broad DOS will produce hybridized states with a single, broad distribution. In the tight binding linear combination of molecular orbitals picture, the states that make up both the L and Σ points of the Brillouin zone consist of hybridized Pb 6p orbitals (mostly the conduction band) and S 4p orbitals (mostly the valence band). The bonding between Pb and S has contributions from both ppσ and ppπ bonds. The minimum at both the L and Σ directions occurs due to the bonding configuration of the linear combination of orbitals in the L and Σ directions. At the NC surface, these p-orbitals will protrude from the surface and be able to participate in bonding with surface-bound molecules. Thus, the hybrid B states arise from the parallel Tc(Ac-COOH)_2_ geometry as the proximity of the molecular π-system to the QD surface which allows it to interact with PbS p-orbitals that make up the 1S(Σ) states,^62,63^ and are directed out-of-plane from the surface, while very little interaction occurs with the 1S(L) states.

## Conclusion

We have demonstrated that the binding of Tc(Ac-COOH)_2_ to PbS QD surfaces produces strong electronic coupling and hybrid states that depend on (1) the nature of the PbS states involved and (2) the molecular orientation of surface-anchored species. Electronic coupling of the diacid and QD produces two types of hybrid states: a narrow split band when the π-system is in a tilted geometry and a broad band from less coupled QD–ligand states when it is in an upright geometry, with respect to the surface. The more strongly coupled hybrid state has a splitting that is reflected in the UV/vis absorption spectra. This strong coupling between the Tc(Ac-COOH)_2_ and PbS is similar to numerous reports concerning flat-lying organic dyes, where the delocalized π-system is parallel and close to the semiconductor/metallic surface. Importantly, different regimes of electronic coupling are associated with different structures of the same ligand, which is the result of employing a bifunctional ligand with a predisposition toward parallel bonding that affects both the electronic and optical properties of the hybrid system. This study highlights that it is crucial to determine the interfacial structure of these systems preceding interpretation of the excited state optical properties.

## Methods

### Materials

#### QD Synthesis and Film Preparation

Lead oxide (99.9995%, Puratronic) and 1-dodecylamine (98%) were purchased from Alfa Aesar. Oleic acid (90%), octane (anhydrous, ≥99%), acetonitrile (>99.9%), trifluoroacetic acid (99%), trifluoroacetic anhydride (≥99%), triethylamine (≥99.5%), toluene (≥99.5%), methyl acetate (anhydrous, 99.5%), tetrahydrofuran (THF, anhydrous, ≥99.9%), tetrachloroethylene (anhydrous, ≥99%), deuterated benzene (anhydrous, ≥99.6%), deuterated chloroform (anhydrous, ≥99.8%), dichloromethane (DCM, anhydrous, ≥99.8%), ferrocene (98%), isopropyl isothiocyanate (97%), 2-isopropylaniline (97%), phenyl isothiocyanate (98%), 4-trifluoromethylcinnamic acid (99%), and 4-dimethylaminocinnamic acid (99%) were purchased from Sigma-Aldrich. Standard grade, uncoated, 2 mm calcium fluoride IR windows were purchased from Knight Optical.

PbS QDs were synthesized under oxygen- and water-free conditions using a synthesis developed by Hendricks et al.^64^ For the synthesis of 2.8 nm PbS/OA, 2.125 g of Pb(oleate)_2_ was added to 37 mL of octane in a 100 mL two-neck flask with an air-free valve. In a 20 mL scintillation vial, 0.417 g of *N*′,*N*′-diphenylthiourea was added with 1.25 mL of diglyme. Both solutions were brought to 90 °C for 15 min with stirring and under a N_2_ flow. During this time, the respective solids dissolved, and then the entire thiourea solution was rapidly injected into the Pb(oleate)_2_ solution. The reaction was removed from heat after ∼60 s, and the product was cooled to RT and dried under vacuum for >1 h. Once dried, the flask and contents were transferred to a nitrogen-filled glovebox, dissolved in 20 mL of toluene, and centrifuged at 8000 rpm for 10 min, followed by 4–6 cycles of precipitation/centrifugation with hexane (solvent) and methyl acetate (antisolvent). The samples were stored in a nitrogen-filled glovebox.

#### Ligand Synthesis

See SI section 7 in the Supporting Information.

#### Ligand Exchange

All film depositions and solid-state ligand exchange reactions were performed in a nitrogen-filled glovebox.

##### QD Films

PbS/oleate QD films were deposited onto silicon (for GIWAXS/GISAXS) and CaF_2_ substrates (for FTIR and UV–vis transmission). Prior to film deposition, the substrates were cleaned by sonicating in isopropyl alcohol for 5 min and then in acetone for 5 min, followed by drying under a gentle nitrogen stream. The QD film deposition solution was made by dissolving dry QDs in octane at a concentration of 70–75 mg/mL. In a spin coater in a glovebox, the QD solution was dropped onto the substrate only to completely cover the substrate and then spun at 1500 rpm for 30 s. The resulting QD films were dried in the glovebox chamber for 5–10 min to remove any remaining octane.

##### Ligand Solutions

Solutions of Tc(Ac-COOH)_2_ were made by dissolving the solid powder in DMF; the solid readily dissolved in the solvent. The solutions were made from material prepared within 6 months of the ligand exchange experiment.

##### Ligand Exchange

The PbS/oleate films were placed in a Tc(Ac-COOH)_2_ solution for 4 h, then rinsed briefly with DMF followed by THF, and dried under vacuum before taking measurements.

#### Spectroscopic Methods

UV/vis/NIR absorption was measured on a Cary 5000 instrument using a background spectrum of a clean CaF_2_ window for spectra in Figure 2a–h. For the powder spectrum of neat Tc(Ac-COOH)_2_, diffuse reflectance infrared Fourier transform spectroscopy (DRIFTS) was performed using a Bruker Alpha FTIR spectrometer in an argon-filled glovebox, and the sample was applied to a gold-coated Si wafer. Baselines were corrected by selecting points on the spectrum where no peaks were present and fitting the baseline using the Bruker FTIR software package. A Coherent Libra Ti:sapphire laser with a repetition rate of 1 kHz and a fundamental wavelength of 800 nm (100 fs pulse width) was used for ultrafast transient absorption experiments. The 550 nm (20 nJ/pulse) pump pulses were generated in an optical parametric amplifier (TOPAS-C, Light Conversion). The probe pulse (λ probe = 400 nm to 1650 nm) was generated by focusing a small portion of the Libra output into a sapphire crystal. The probe pulse was focused at the sample and pump, probe pulses were spatially overlapped, and a mechanical delay stage was used to delay the probe pulse relative to the pump. The time window for the experiment is 5 ns. A small portion of the probe was redirected before the sample to be used as a reference to reduce noise. Changes in the probe spectrum were monitored through a fiber optic coupled multichanneled spectrometer with a CMOS sensor. Helios and Surface Xplorer software from Ultrafast Systems were used to collect and chirp-correct the data, respectively.

#### Grazing Incidence X-ray Scattering

See section 5 in the Supporting Information.

## Supporting Information Available

UV–vis, FTIR, TA, GIWAXS/GISAXS, and DFT data, as well as synthesis information for the dicarboxylic-functionalized tetracene ligand (PDF)

## References

[ref1] Hanna M. C.; Nozik A. J. Solar Conversion Efficiency of Photovoltaic and Photoelectrolysis Cells with Carrier Multiplication Absorbers. J. Appl. Phys. 2006, 100, 074510 10.1063/1.2356795.

[ref2] Shockley W.; Queisser H. J. Detailed Balance Limit of Efficiency of *P-n* Junction Solar Cells. J. Appl. Phys. 1961, 32, 510–519. 10.1063/1.1736034.

[ref3] Gholizadeh E. M.; Prasad S. K. K.; Teh Z. L.; Ishwara T.; Norman S.; Petty A. J.; Cole J. H.; Cheong S.; Tilley R. D.; Anthony J. E.; Huang S.; Schmidt T. W. Photochemical Upconversion of Near-Infrared Light from below the Silicon Bandgap. Nat. Photonics 2020, 14, 585–590. 10.1038/s41566-020-0664-3.

[ref4] De Roo J.; Huang Z.; Schuster N. J.; Hamachi L. S.; Congreve D. N.; Xu Z.; Xia P.; Fishman D. A.; Lian T.; Owen J. S.; Tang M. L. Anthracene Diphosphate Ligands for CdSe Quantum Dots; Molecular Design for Efficient Upconversion. Chem. Mater. 2020, 32, 1461–1466. 10.1021/acs.chemmater.9b04294.

[ref5] Papa C. M.; Garakyaraghi S.; Granger D. B.; Anthony J. E.; Castellano F. N. TIPS-Pentacene Triplet Exciton Generation on PbS Quantum Dots Results from Indirect Sensitization. Chemical Science 2020, 11, 5690–5696. 10.1039/D0SC00310G. 32864083 PMC7425078

[ref6] Huang Z.; Xu Z.; Mahboub M.; Liang Z.; Jaimes P.; Xia P.; Graham K. R.; Tang M. L.; Lian T. Enhanced Near-Infrared-to-Visible Upconversion by Synthetic Control of PbS Nanocrystal Triplet Photosensitizers. J. Am. Chem. Soc. 2019, 141, 9769–9772. 10.1021/jacs.9b03385. 31180212

[ref7] Mahboub M.; Huang Z.; Tang M. L. Efficient Infrared-to-Visible Upconversion with Subsolar Irradiance. Nano Lett. 2016, 16, 7169–7175. 10.1021/acs.nanolett.6b03503. 27788577

[ref8] Lu H.; Chen X.; Anthony J. E.; Johnson J. C.; Beard M. C. Sensitizing Singlet Fission with Perovskite Nanocrystals. J. Am. Chem. Soc. 2019, 141, 4919–4927. 10.1021/jacs.8b13562. 30821456

[ref9] Zhang J.; Sakai H.; Suzuki K.; Hasobe T.; Tkachenko N. V.; Chang I.-Y.; Hyeon-Deuk K.; Kaji H.; Teranishi T.; Sakamoto M. Near-Unity Singlet Fission on a Quantum Dot Initiated by Resonant Energy Transfer. J. Am. Chem. Soc. 2021, 143, 17388–17394. 10.1021/jacs.1c04731. 34647732

[ref10] Huang Z.; Beard M. C. Dye-Sensitized Multiple Exciton Generation in Lead Sulfide Quantum Dots. J. Am. Chem. Soc. 2022, 144, 15855–15861. 10.1021/jacs.2c07109. 35981268 PMC9437916

[ref11] Allardice J. R.; Thampi A.; Dowland S.; Xiao J.; Gray V.; Zhang Z.; Budden P.; Petty A. J.; Davis N. J. L. K.; Greenham N. C.; Anthony J. E.; Rao A. Engineering Molecular Ligand Shells on Quantum Dots for Quantitative Harvesting of Triplet Excitons Generated by Singlet Fission. J. Am. Chem. Soc. 2019, 141, 12907–12915. 10.1021/jacs.9b06584. 31336046 PMC7007228

[ref12] Kroupa D. M.; Arias D. H.; Blackburn J. L.; Carroll G. M.; Granger D. B.; Anthony J. E.; Beard M. C.; Johnson J. C. Control of Energy Flow Dynamics between Tetracene Ligands and PbS Quantum Dots by Size Tuning and Ligand Coverage. Nano Lett. 2018, 18, 865–873. 10.1021/acs.nanolett.7b04144. 29364676

[ref13] Luo X.; Han Y.; Chen Z.; Li Y.; Liang G.; Liu X.; Ding T.; Nie C.; Wang M.; Castellano F. N.; Wu K. Mechanisms of Triplet Energy Transfer across the Inorganic Nanocrystal/Organic Molecule Interface. Nat. Commun. 2020, 11, 1–10. 10.1038/s41467-019-13951-3. 31911606 PMC6946700

[ref14] Bender J. A.; Raulerson E. K.; Li X.; Goldzak T.; Xia P.; Van Voorhis T.; Tang M. L.; Roberts S. T. Surface States Mediate Triplet Energy Transfer in Nanocrystal-Acene Composite Systems. J. Am. Chem. Soc. 2018, 140, 7543–7553. 10.1021/jacs.8b01966. 29846066

[ref15] Kroupa D. M.; Vörös M.; Brawand N. P.; Bronstein N.; McNichols B. W.; Castaneda C. V.; Nozik A. J.; Sellinger A.; Galli G.; Beard M. C. Optical Absorbance Enhancement in PbS QD/Cinnamate Ligand Complexes. J. Phys. Chem. Lett. 2018, 9, 3425–3433. 10.1021/acs.jpclett.8b01451. 29857647

[ref16] Kroupa D. M.; Vörös M.; Brawand N. P.; McNichols B. W.; Miller E. M.; Gu J.; Nozik A. J.; Sellinger A.; Galli G.; Beard M. C. Tuning Colloidal Quantum Dot Band Edge Positions through Solution-Phase Surface Chemistry Modification. Nat. Commun. 2017, 8, 15257 10.1038/ncomms15257. 28508866 PMC5440806

[ref17] Brown P. R.; Kim D.; Lunt R. R.; Zhao N.; Bawendi M. G.; Grossman J. C.; Bulović V. Energy Level Modification in Lead Sulfide Quantum Dot Thin Films through Ligand Exchange. ACS Nano 2014, 8, 5863–5872. 10.1021/nn500897c. 24824726

[ref18] Frederick M. T.; Amin V. A.; Weiss E. A. Optical Properties of Strongly Coupled Quantum Dot-Ligand Systems. J. Phys. Chem. Lett. 2013, 4, 634–640. 10.1021/jz301905n. 26281879

[ref19] Frederick M. T.; Weiss E. A. Relaxation of Exciton Confinement in CdSe Quantum Dots by Modification with a Conjugated Dithiocarbamate Ligand. ACS Nano 2010, 4, 3195–3200. 10.1021/nn1007435. 20503978

[ref20] Giansante C.; Infante I.; Fabiano E.; Grisorio R.; Suranna G. P.; Gigli G. Darker-than-Black” PbS Quantum Dots: Enhancing Optical Absorption of Colloidal Semiconductor Nanocrystals via Short Conjugated Ligands. J. Am. Chem. Soc. 2015, 137, 1875–1886. 10.1021/ja510739q. 25574692

[ref21] Giansante C. Enhancing Light Absorption by Colloidal Metal Chalcogenide Quantum Dots via Chalcogenol(Ate) Surface Ligands. Nanoscale 2019, 11, 9478–9487. 10.1039/C9NR01785B. 31045198

[ref22] Kroupa D. M.; Anderson N. C.; Castaneda C. V.; Nozik A. J.; Beard M. C. In Situ Spectroscopic Characterization of a Solution-Phase X-Type Ligand Exchange at Colloidal Lead Sulphide Quantum Dot Surfaces. Chem. Commun. 2016, 52, 13893–13896. 10.1039/C6CC08114B. 27841383

[ref23] Bronstein N. D.; Martinez M. S.; Kroupa D. M.; Vörös M.; Lu H.; Brawand N. P.; Nozik A. J.; Sellinger A.; Galli G.; Beard M. C. Designing Janus Ligand Shells on PbS Quantum Dots Using Ligand-Ligand Cooperativity. ACS Nano 2019, 13, 3839–3846. 10.1021/acsnano.9b00191. 30855942

[ref24] Martinez M. S.; Nozik A. J.; Beard M. C. Size-Dependent Janus-Ligand Shell Formation on PbS Quantum Dots. J. Phys. Chem. C 2021, 125, 21729–21739. 10.1021/acs.jpcc.1c06713.

[ref25] Harris R. D.; Amin V. A.; Lau B.; Weiss E. A. Role of Interligand Coupling in Determining the Interfacial Electronic Structure of Colloidal CdS Quantum Dots. ACS Nano 2016, 10, 1395–1403. 10.1021/acsnano.5b06837. 26727219

[ref26] Huang Z.; Hao J.; Blackburn J. L.; Beard M. C. Pyroelectricity of Lead Sulfide (PbS) Quantum Dot Films Induced by Janus-Ligand Shells. ACS Nano 2021, 15, 14965–14971. 10.1021/acsnano.1c05185. 34402613

[ref27] Huang Z.; Koubek J. T.; Sellinger A.; Beard M. C. Pickering Emulsions of Self-Assembled Lead Sulfide Quantum Dots with Janus-Ligand Shells as Nanoreactors for Photocatalytic Reactions. ACS Appl. Nano Mater. 2022, 5, 3183–3187. 10.1021/acsanm.2c00341.

[ref28] Muscat J. P.; Newns D. M. Chemisorption on Metals. Prog. Surf. Sci. 1978, 9, 1–43. 10.1016/0079-6816(78)90005-9.

[ref29] Persson P.; Lundqvist M. J.; Nilsing M.; van Duin A. C.T.; Goddard W. A. III In Quantum-Chemical Calculations of Dye-Sensitized Semiconductor Nanocrystals; Spitler M., Willig F., Eds.; SPIE: 2006; p 63250P. 10.1021/ct050141x 26626531

[ref30] Lasser L.; Ronca E.; Pastore M.; De Angelis F.; Cornil J.; Lazzaroni R.; Beljonne D. Energy Level Alignment at Titanium Oxide-Dye Interfaces: Implications for Electron Injection and Light Harvesting. J. Phys. Chem. C 2015, 119, 9899–9909. 10.1021/acs.jpcc.5b01267.

[ref31] Greiner M. T.; Jones T. E.; Beeg S.; Zwiener L.; Scherzer M.; Girgsdies F.; Piccinin S.; Armbrüster M.; Knop-Gericke A.; Schlögl R. Free-Atom-like d States in Single-Atom Alloy Catalysts. Nature Chem. 2018, 10, 1008–1015. 10.1038/s41557-018-0125-5. 30150725

[ref32] Moreels I.; Lambert K.; Smeets D.; De Muynck D.; Nollet T.; Martins J. C.; Vanhaecke F.; Vantomme A.; Delerue C.; Allan G.; Hens Z. Size-Dependent Optical Properties of Colloidal PbS Quantum Dots. ACS Nano 2009, 3, 3023–3030. 10.1021/nn900863a. 19780530

[ref33] Kang I.; Wise F. W. Electronic Structure and Optical Properties of PbS and PbSe Quantum Dots. J. Opt. Soc. Am. B, JOSAB 1997, 14, 1632–1646. 10.1364/JOSAB.14.001632.

[ref34] Miller E. M.; Kroupa D. M.; Zhang J.; Schulz P.; Marshall A. R.; Kahn A.; Lany S.; Luther J. M.; Beard M. C.; Perkins C. L.; van de Lagemaat J. Revisiting the Valence and Conduction Band Size Dependence of PbS Quantum Dot Thin Films. ACS Nano 2016, 10, 3302–3311. 10.1021/acsnano.5b06833. 26895310

[ref35] Kennehan E. R.; Doucette G. S.; Marshall A. R.; Grieco C.; Munson K. T.; Beard M. C.; Asbury J. B. Electron-Phonon Coupling and Resonant Relaxation from 1D and 1P States in PbS Quantum Dots. ACS Nano 2018, 12, 6263–6272. 10.1021/acsnano.8b03216. 29792675

[ref36] Hoertz P. G.; Carlisle R. A.; Meyer G. J.; Wang D.; Piotrowiak P.; Galoppini E. Organic Rigid-Rod Linkers for Coupling Chromophores to Metal Oxide Nanoparticles. Nano Lett. 2003, 3, 325–330. 10.1021/nl025946g.

[ref37] Taratula O.; Rochford J.; Piotrowiak P.; Galoppini E.; Carlisle R. A.; Meyer G. J. Pyrene-Terminated Phenylenethynylene Rigid Linkers Anchored to Metal Oxide Nanoparticles. J. Phys. Chem. B 2006, 110, 15734–15741. 10.1021/jp0623847. 16898719

[ref38] Ernstorfer R.; Gundlach L.; Felber S.; Storck W.; Eichberger R.; Willig F. Role of Molecular Anchor Groups in Molecule-to-Semiconductor Electron Transfer. J. Phys. Chem. B 2006, 110, 25383–25391. 10.1021/jp064436y. 17165985

[ref39] Johnston E. E.; Trammell S. A.; Goldston H. M.; Conrad D. W. Sensitization of Nanoporous TiO2 Electrodes Using the Naturally Occurring Chromophores: Stentorin and Hypericin. J. Photochem. Photobiol., A 2001, 140, 179–183. 10.1016/S1010-6030(01)00406-3.

[ref40] Rochford J.; Chu D.; Hagfeldt A.; Galoppini E. Tetrachelate Porphyrin Chromophores for Metal Oxide Semiconductor Sensitization: Effect of the Spacer Length and Anchoring Group Position. J. Am. Chem. Soc. 2007, 129, 4655–4665. 10.1021/ja068218u. 17385856

[ref41] Rochford J.; Galoppini E. Zinc(II) Tetraarylporphyrins Anchored to TiO2, ZnO, and ZrO2 Nanoparticle Films through Rigid-Rod Linkers. Langmuir 2008, 24, 5366–5374. 10.1021/la703845u. 18410135

[ref42] Kennehan E. R.; Munson K. T.; Doucette G. S.; Marshall A. R.; Beard M. C.; Asbury J. B. Dynamic Ligand Surface Chemistry of Excited PbS Quantum Dots. J. Phys. Chem. Lett. 2020, 11, 2291–2297. 10.1021/acs.jpclett.0c00539. 32131595

[ref43] Deacon G. B.; Phillips R. J. Relationships between the Carbon-Oxygen Stretching Frequencies of Carboxylato Complexes and the Type of Carboxylate Coordination. Coord. Chem. Rev. 1980, 33, 227–250. 10.1016/S0010-8545(00)80455-5.

[ref44] Cass L. C.; Malicki M.; Weiss E. A. The Chemical Environments of Oleate Species within Samples of Oleate-Coated PbS Quantum Dots. Anal. Chem. 2013, 85, 6974–6979. 10.1021/ac401623a. 23786216

[ref45] Long R.; Casanova D.; Fang W.-H.; Prezhdo O. V. Donor-Acceptor Interaction Determines the Mechanism of Photoinduced Electron Injection from Graphene Quantum Dots into TiO2: π-Stacking Supersedes Covalent Bonding. J. Am. Chem. Soc. 2017, 139, 2619–2629. 10.1021/jacs.6b09598. 28125783

[ref46] Baumgardner W. J.; Whitham K.; Hanrath T. Confined-but-Connected Quantum Solids via Controlled Ligand Displacement. Nano Lett. 2013, 13, 3225–3231. 10.1021/nl401298s. 23777454

[ref47] Greenler R. G.; Snider D. R.; Witt D.; Sorbello R. S. The Metal-Surface Selection Rule for Infrared Spectra of Molecules Adsorbed on Small Metal Particles. Surf. Sci. 1982, 118, 415–428. 10.1016/0039-6028(82)90197-2.

[ref48] Beygi H.; Sajjadi S. A.; Babakhani A.; Young J. F.; van Veggel F. C. J. M. Surface Chemistry of As-Synthesized and Air-Oxidized PbS Quantum Dots. Appl. Surf. Sci. 2018, 457, 1–10. 10.1016/j.apsusc.2018.06.152.

[ref49] Grisorio R.; Debellis D.; Suranna G. P.; Gigli G.; Giansante C. The Dynamic Organic/Inorganic Interface of Colloidal PbS Quantum Dots. Angew. Chem., Int. Ed. 2016, 55, 6628–6633. 10.1002/anie.201511174. 27038221

[ref50] Sukharevska N.; Bederak D.; Goossens V. M.; Momand J.; Duim H.; Dirin D. N.; Kovalenko M. V.; Kooi B. J.; Loi M. A. Scalable PbS Quantum Dot Solar Cell Production by Blade Coating from Stable Inks. ACS Appl. Mater. Interfaces 2021, 13, 5195–5207. 10.1021/acsami.0c18204. 33470785 PMC7863069

[ref51] Du L.; Tang S.; Hansen A. S.; Frandsen B. N.; Maroun Z.; Kjaergaard H. G. Subtle Differences in the Hydrogen Bonding of Alcohol to Divalent Oxygen and Sulfur. Chem. Phys. Lett. 2017, 667, 146–153. 10.1016/j.cplett.2016.11.045.

[ref52] Coates J. Interpretation of Infrared Spectra, A Practical Approach. In Encyclopedia of Analytical Chemistry; Meyers R. A., Ed.; Wiley: 2006; p a5606.

[ref53] Auer B.; Kumar R.; Schmidt J. R.; Skinner J. L. Hydrogen Bonding and Raman, IR, and 2D-IR Spectroscopy of Dilute HOD in Liquid D2O. Proc. Natl. Acad. Sci. U. S. A. 2007, 104, 14215–14220. 10.1073/pnas.0701482104. 17576923 PMC1964876

[ref54] Nakamoto K.; Margoshes M.; Rundle R. E. Stretching Frequencies as a Function of Distances in Hydrogen Bonds. J. Am. Chem. Soc. 1955, 77, 6480–6486. 10.1021/ja01629a013.

[ref55] Choi J. J.; Bealing C. R.; Bian K.; Hughes K. J.; Zhang W.; Smilgies D.-M.; Hennig R. G.; Engstrom J. R.; Hanrath T. Controlling Nanocrystal Superlattice Symmetry and Shape-Anisotropic Interactions through Variable Ligand Surface Coverage. J. Am. Chem. Soc. 2011, 133, 3131–3138. 10.1021/ja110454b. 21306161

[ref56] Weidman M. C.; Yager K. G.; Tisdale W. A. Interparticle Spacing and Structural Ordering in Superlattice PbS Nanocrystal Solids Undergoing Ligand Exchange. Chem. Mater. 2015, 27, 474–482. 10.1021/cm503626s.

[ref57] Whitham K.; Smilgies D.-M.; Hanrath T. Entropic, Enthalpic, and Kinetic Aspects of Interfacial Nanocrystal Superlattice Assembly and Attachment. Chem. Mater. 2018, 30, 54–63. 10.1021/acs.chemmater.7b04223.

[ref58] Bian K.; Li R.; Fan H. Controlled Self-Assembly and Tuning of Large PbS Nanoparticle Supercrystals. Chem. Mater. 2018, 30, 6788–6793. 10.1021/acs.chemmater.8b02691.

[ref59] Weidman M. C.; Nguyen Q.; Smilgies D.-M.; Tisdale W. A. Impact of Size Dispersity, Ligand Coverage, and Ligand Length on the Structure of PbS Nanocrystal Superlattices. Chem. Mater. 2018, 30, 807–816. 10.1021/acs.chemmater.7b04322.

[ref60] Wang K.; Cline R. P.; Schwan J.; Strain J. M.; Roberts S. T.; Eaves J. D.; Tang M. L. Efficient Photon Upconversion Enabled by Strong Coupling Between Organic Molecules and Quantum Dots. Nat. Chem. 2023, 10.1038/s41557-023-01225-x. 37308710

[ref61] Fujisawa J.; Hanaya M. Extremely Strong Organic-Metal Oxide Electronic Coupling Caused by Nucleophilic Addition Reaction. Phys. Chem. Chem. Phys. 2015, 17, 16285–16293. 10.1039/C5CP01817J. 26041649

[ref62] Kane R. S.; Cohen R. E.; Silbey R. Theoretical Study of the Electronic Structure of PbS Nanoclusters. J. Phys. Chem. 1996, 100, 7928–7932. 10.1021/jp952869n.

[ref63] Brod M. K.; Toriyama M. Y.; Snyder G. J. Orbital Chemistry That Leads to High Valley Degeneracy in PbTe. Chem. Mater. 2020, 32, 9771–9779. 10.1021/acs.chemmater.0c03740.

[ref64] Hendricks M. P.; Campos M. P.; Cleveland G. T.; Jen-La Plante I.; Owen J. S. A Tunable Library of Substituted Thiourea Precursors to Metal Sulfide Nanocrystals. Science 2015, 348, 1226–1230. 10.1126/science.aaa2951. 26068846

