# Supplementary material for: Controlling Electronic Coupling of Acene Chromophores on Quantum Dot Surfaces through Variable-Concentration Ligand Exchange
Source: ACS Nano. 2023 Jul 26;17(15):14916–29. doi: 10.1021/acsnano.3c03498 (PMC10416565; doi:10.1021/acsnano.3c03498)
Supplement: Supplementary file 1 — nn3c03498_si_001.pdf [file nn3c03498_si_001.pdf]

Supporting Information For

Controlling Electronic Coupling of Acene Chromophores on Quantum Dot Surfaces through Variable Concentration Ligand Exchange

Marissa S. Martinez[1], Michelle Nolen[2], Nicholas Pompetti[3], Lee J. Richter[4], Carrie A. Farberow[5], Justin C. Johnson[1*], and Matthew C. Beard[1*]

[1] Chemistry & Nanoscience Center, National Renewable Energy Laboratory, Golden, Colorado 80401, United States

[2] Department of Chemical and Biological Engineering, Colorado School of Mines, Golden, Colorado 80401, United States

[3] Department of Chemistry, University of Colorado Boulder, Boulder, Colorado 80309, United States

[4] Materials Science and Engineering Division, National Institute of Standards and Technology, Gaithersburg, Md 20899, USA

[5] Catalytic Carbon Transformation & Scale-Up Center, National Renewable Energy Laboratory, Golden, Colorado 80401, United States

Contents:

1. UV-VIS Data…2
2. DFT methods…3
3. Experimental FTIR…7
4. Table of FTIR peak assignments…11
5. Small and Wide Angle X-ray Scattering…12
6. Transient absorption of neat ligand in solution…18
7. Ligand synthesis and characterization…19

1. UV-VIS Data

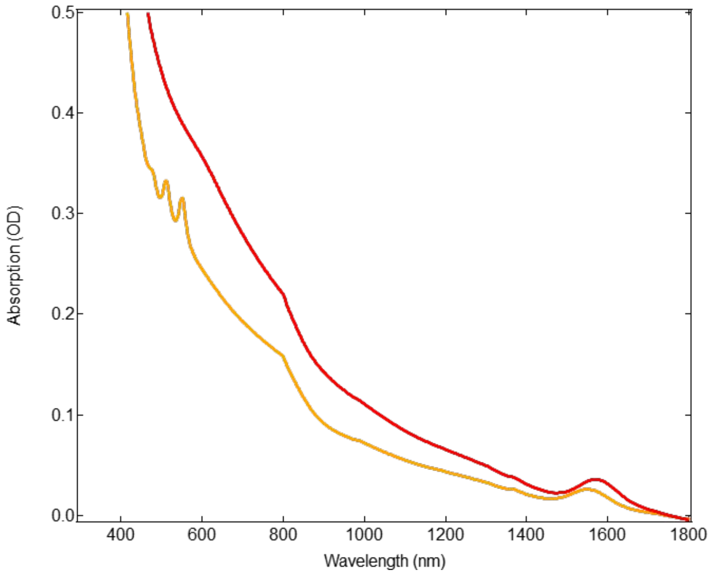

Figure S1. Absorption spectra of PbS QD films after treatment with 4 mmol/L of the TIPS-Tc-COOH acid (orange) and TIPS-Tc-$(Ac)_2$COOH (monoacid version, red).

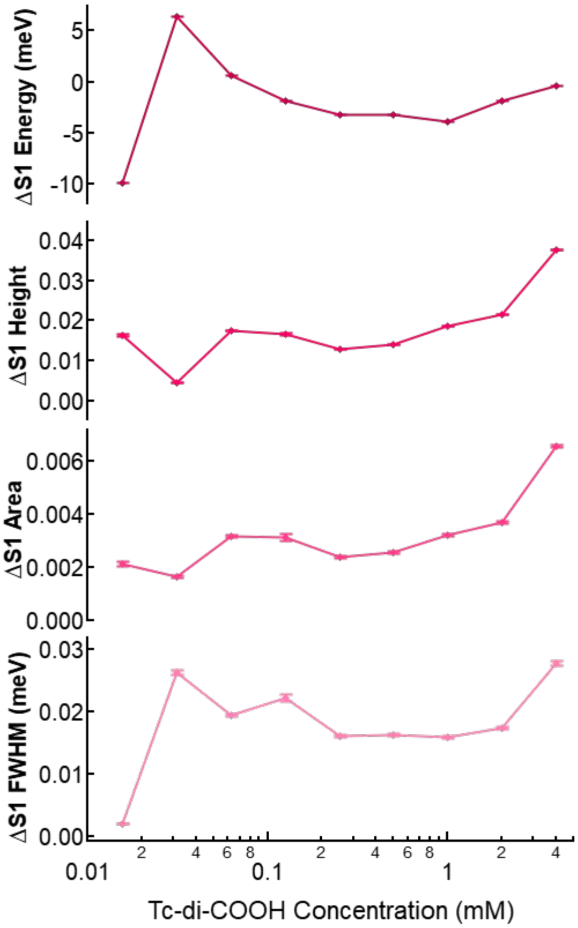

Figure S2. Parameters from fitting QD first exciton absorption peak in Tc-di-COOH treatment series.

2. DFT Methods

DFT calculations were performed using the Vienna Ab initio Simulation Package (VASP).[1] The generalized gradient approximation (GGA) with the Perdew-Burke-Ernzerhof (PBE) functional[2] was used to approximate the exchange-correlation energy functional. The DFT-D3 method of Grimme et al.[3] was applied to account for dispersion forces. Electron-ion interactions were described using the projector augmented-wave (PAW) method[4] with electronic wave functions expanded in a plane-wave basis set with a kinetic energy cutoff of 400 eV. The electronic convergence criterion was set to 10^{-6} eV between successive electronic relaxation steps, while ionic convergence was set to occur when the Hellmann-Feynman forces acting upon each atom fell below 0.02 eV/Å.

The PbS(100) and PbS(111) surfaces were modelled as slabs with six atomic layers. The three and four uppermost atomic layers were allowed to relax on the (100) and (111) surfaces, respectively. Prior reports have indicated that 6 - 8 atomic layers are sufficient to capture the behavior of the PbS surface,[5] and that bulk-like behavior appears after four atomic layers.[6] We found that 6 atomic-layer slabs were sufficient in capturing the trends in the adsorption modes of tetracene on the (111) and (100) facets, while balancing computational cost. The calculated lattice parameter is 5.958Å, in good agreement with experimentally reported values.[7] The (111) and (100) PbS surfaces were modelled with respective 5×7×6 and 6×8×6 periodic unit cells in x, y, and z directions (Figure. S3), with at least 20 Å of vacuum between successive slabs in the *z* direction. The Monkhorst-Pack scheme[8] of 2×2×1 was used for sampling of the Brillouin zone. Adsorption energies of tetracene were calculated by:

$$E_{ads} = E_{adsorbed\ tetracene} - E_{PbS} - E_{gas\text{-}phase\ tetracene} \quad (01)$$

Where Eadsorbed tetracene is the electronic energy of the total system, including PbS and the adsorbed molecule, E_{PbS} is the energy of the clean PbS slab of the appropriate facet, and $E_{gas\text{-}phase\ tetracene}$ is the gas-phase energy of a tetracene diacid molecule with one deprotonated COOH group.

Vibrational frequencies were calculated using a finite-difference approximation of the Hessian matrix[9] where atom locations were perturbed by a displacement of 0.015 Å in the x, y, and z directions. All bound intermediates were fully relaxed during the vibrational analysis, while all PbS atoms remained fixed. The intensity of vibrational modes was determined by analyzing the OUTCAR from a vibrational calculation with a code developed for this purpose.[10] The relative infrared intensity of each vibrational mode (*i*) were approximated by taking the square of the directional derivative of the dipole moment ($\partial\mu_z/\partial Q_i$), where μ_z is the dipole moment in the z direction (direction perpendicular to the PbS slab) and Q_i is the distance of perturbation for each mode.[11] Lorentzian broadening with an arbitrary width of 20 cm^{-1} was applied. Charge density difference plots were generated in VESTA with CHGCARs from single-point calculations.

Table 1. Adsorption energies (eV) of vertical, tilted, and parallel modes of tetracene diacid on PbS(111) and PbS(100).

Surface	*Vertical (× 2)*	*Tilted (× 2)*	*Parallel*
PbS(111)	-3.92 (-7.85)		-6.58
PbS(100)	-1.84 (-3.69)		-3.21

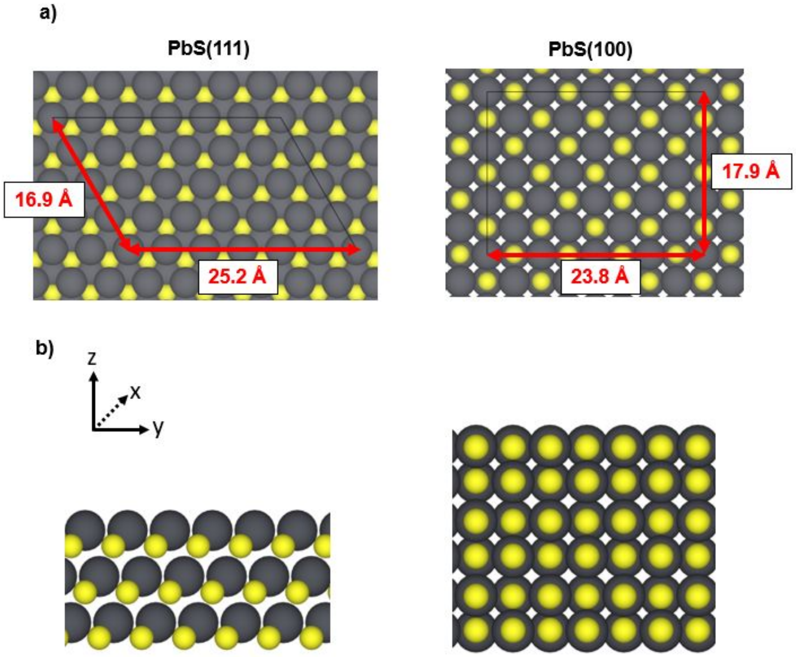

Figure S3. Snapshots of PbS(100) and PbS(111) a) top views and b) side views of slab models used to simulate PbS surfaces. Unit cells are indicated by the black line in the top view with their dimensionalities marked in red.

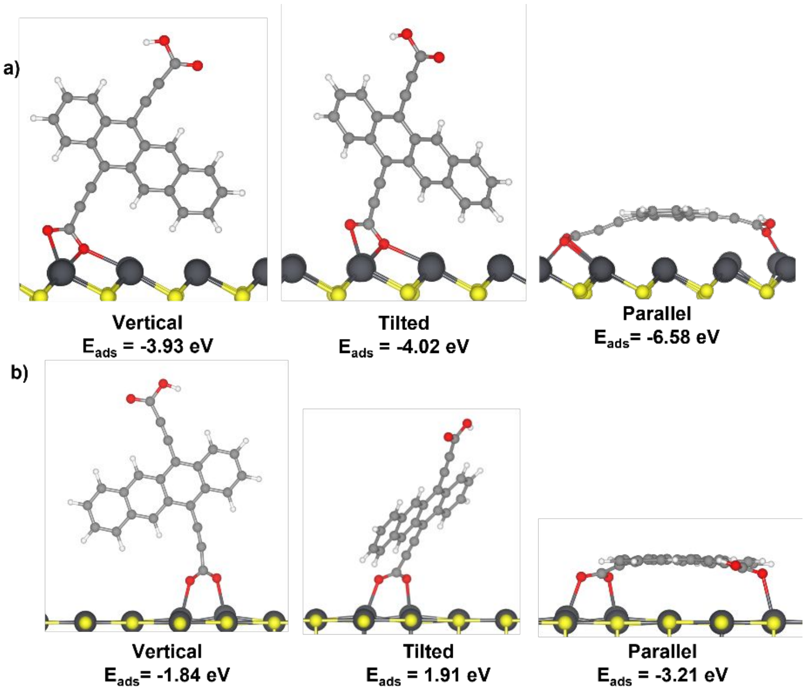

Figure S4. Side views of adsorbed tetracene a) PbS(111) and b) PbS(100) for vertical, tilted, and parallel adsorption modes.

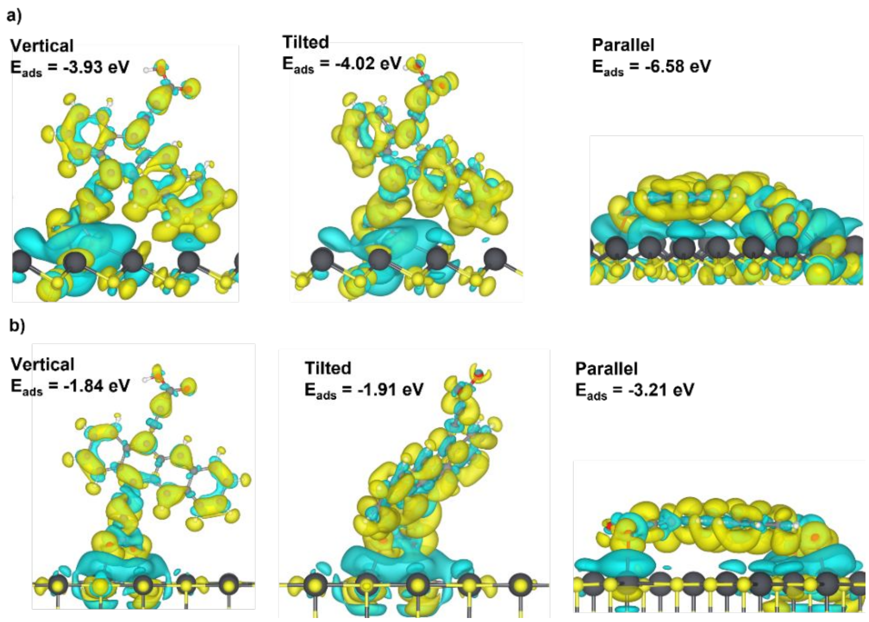

Figure S5. Side views of charge density difference plots on a) PbS(111) and b) PbS(100) for both the vertical, tilted, and parallel adsorption modes of tetracene. Yellow indicates an increase in electron density, and blue indicates a depletion.

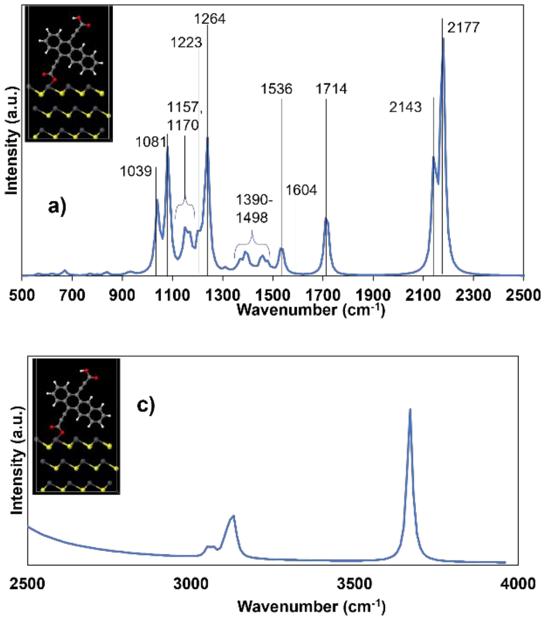

Figure S6. Calculated IR frequencies of the vertical (a,c) and parallel (b,d) Tc(Ac-COOMe)$_2$ geometries on the PbS (111) facets.

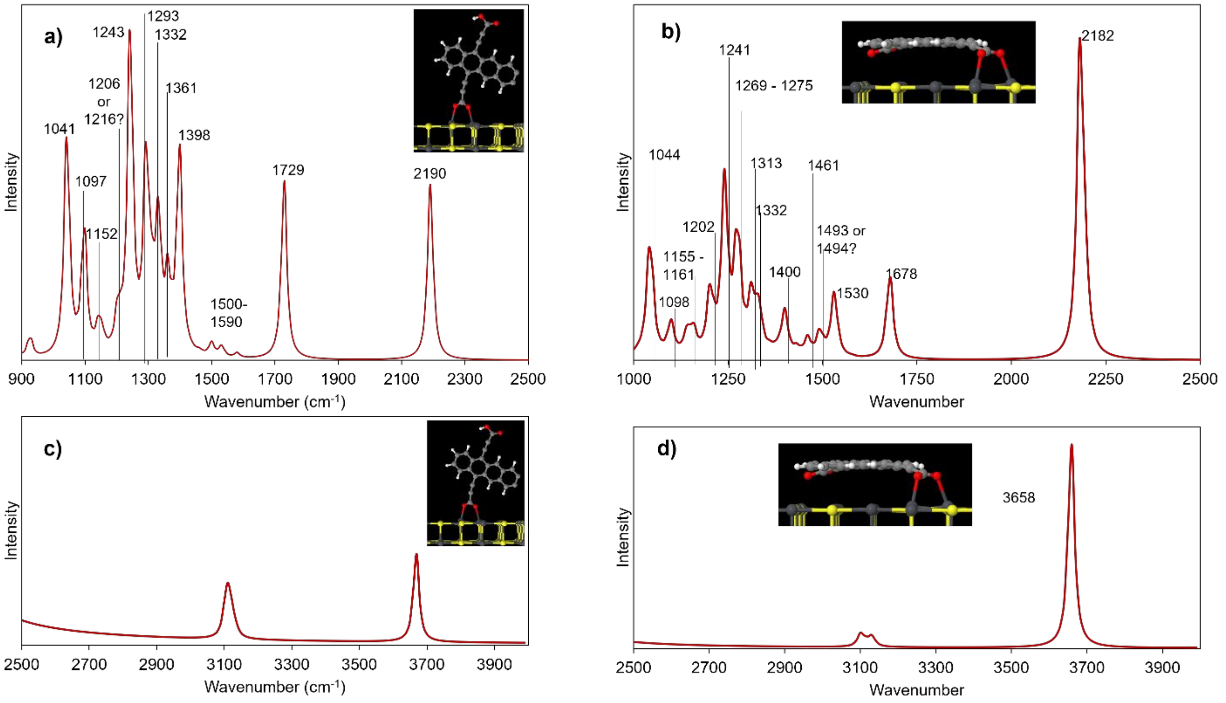

Figure S7. Calculated IR frequencies of the vertical (a,c) and parallel (b,d) Tc(Ac-COOMe)$_2$ geometries on the PbS (100) facets.

3. FTIR Spectra

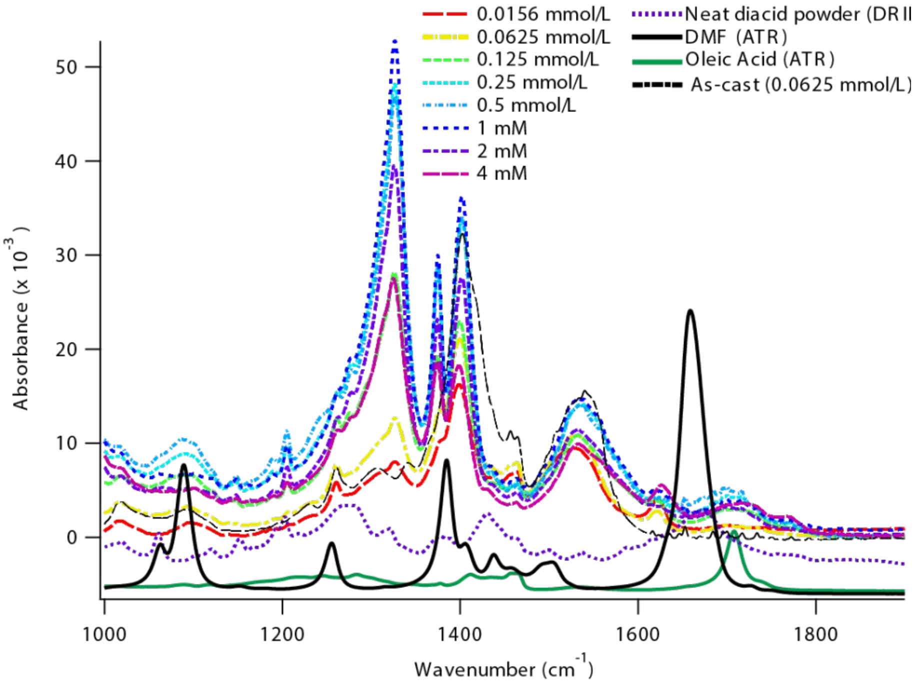

Figure S8. Transmission-FTIR carboxylate spectra of Tc(Ac-COOMe)$_2$ treatment series

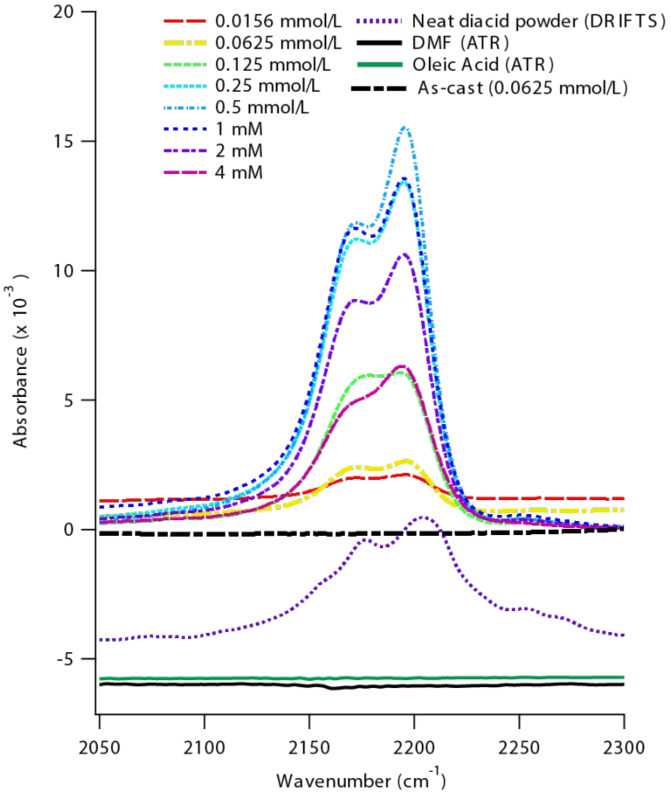

Figure S9. Transmission-FTIR alkyne spectra of Tc(Ac-COOMe)$_2$ treatment series.

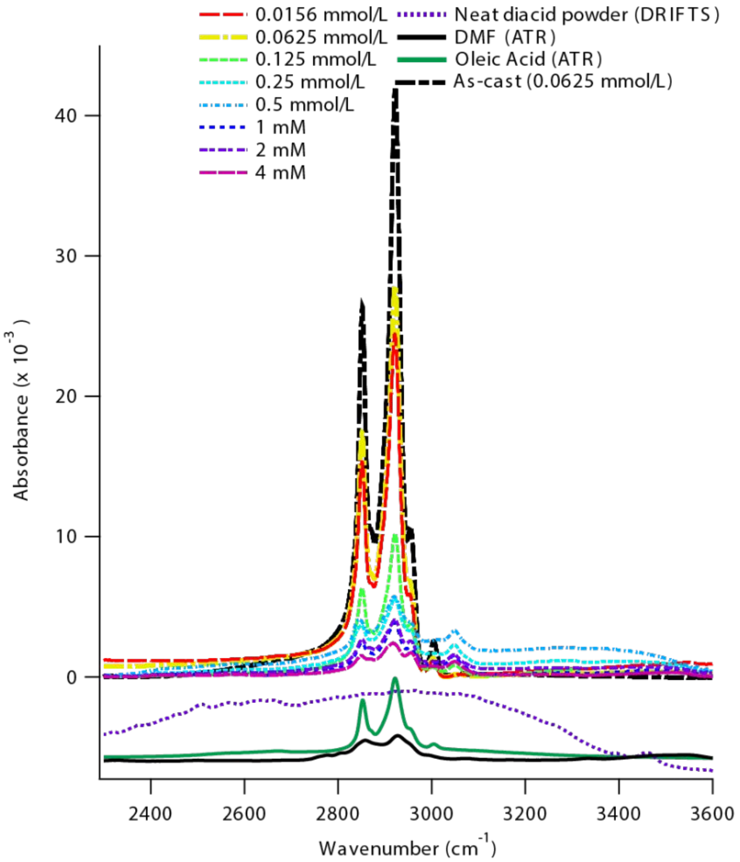

Figure S10. Transmission-FTIR aliphatic spectra of $Tc(Ac\text{-}COOH)_2$ treatment series.

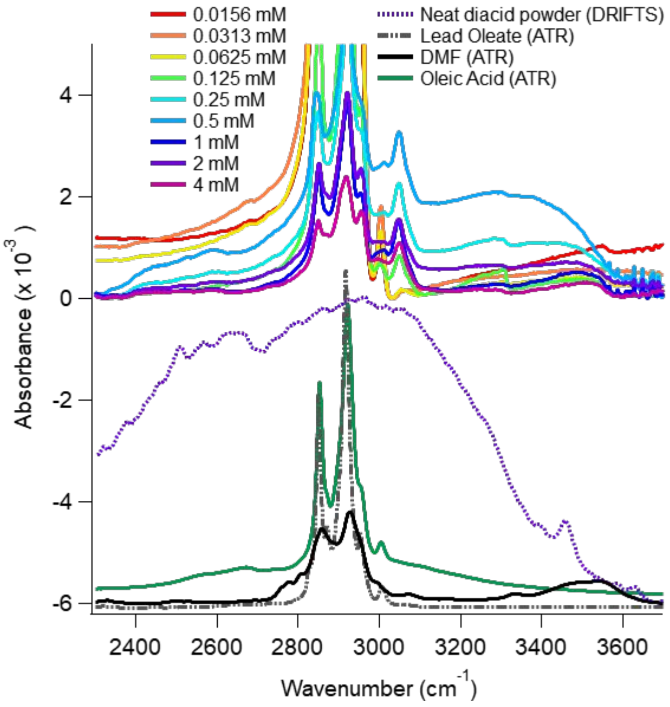

Figure S11. Transmission-FTIR hydroxyl spectra of $Tc(Ac\text{-}COOH)_2$ treatment series.

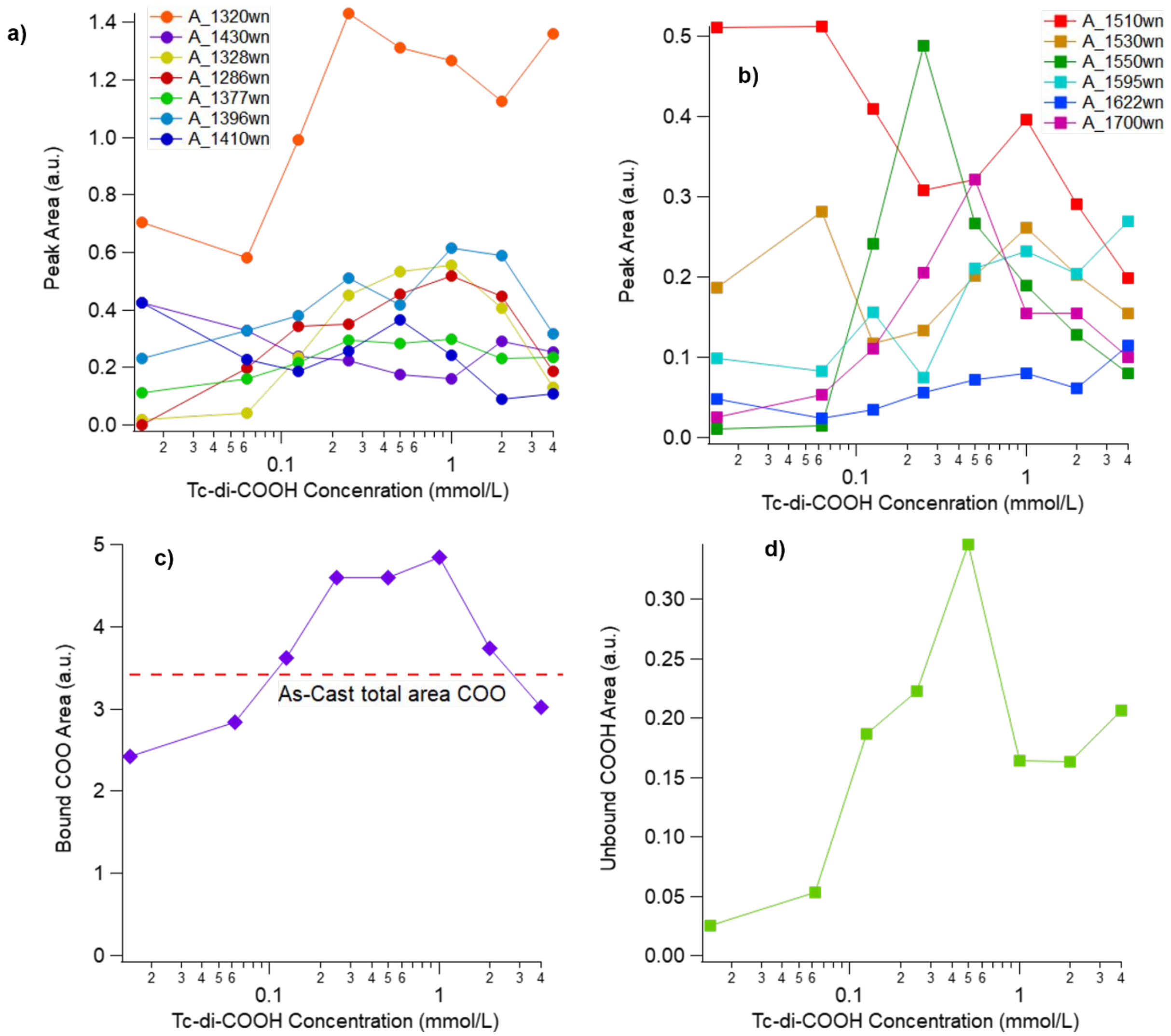

Figure S12. Carboxylate peak peak areas for bound COO (left) and unbound C=O (right).

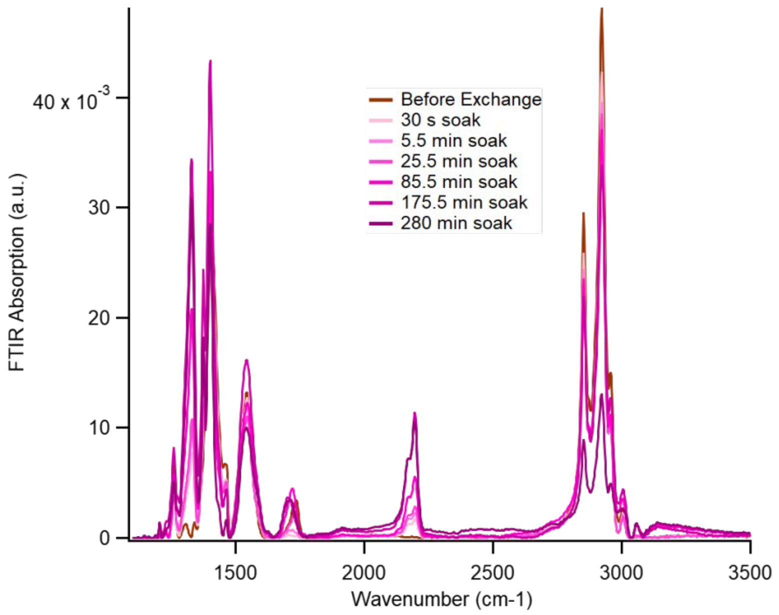

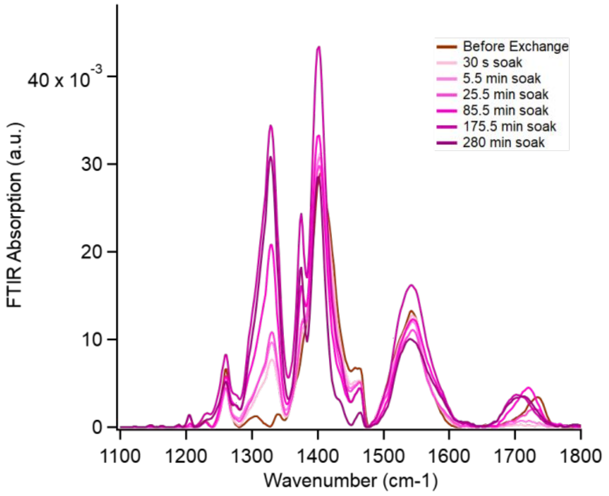

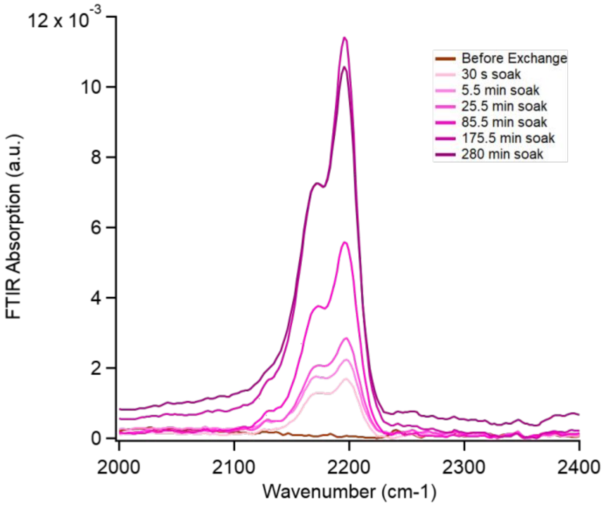

Figure S13. Soaking time-dependent FTIR spectra using 0.5 mmol/L Tc(Ac-COOH)$_2$ solution.

Table S2. FTIR Peak Assignments for the As-Cast, PbS/Tc-di-COOH, Neat Tc-di-COOH powder, and Drop Cast Methyl Ester

ν (cm^{-1})	Assignment	Label in Text
	Methyl Ester	
1063		-
1147		-
1201		-
1244	C-O	-
1370		-
1396		-
1434		-
1716	C=O	-
2203	C≡C	-
2820-3085	C-H	-
	Di-Acid	
1018		-
1061		-
1120		-
1152		-
1203		-
1265-1281		-
1320		-
1377		-
1430		-
1500		-
1536		-
1665-1675	C=O	-
2177	C≡C	-
2204	C≡C	-
2300-3660	C-H, -OH (H-bonded)	-
	QD Films - post treatment	
1286	C-O-H	4
1320	COO^{-}_{uni}, sym**	3
1328	COO^{-}_{uni}, sym	2
1377	COO^{-}_{bridge}, sym**	1c
1398	COO^{-}_{bridge}, sym	1b
1407	COO^{-}_{bridge}, sym	1a
1435	Ring	
1510	COO^{-}_{bridge}, asym	1a
1531	COO^{-}_{bridge}, asym	1b
1550	COO^{-}_{bridge}, asym	1c
1590	COO^{-}_{uni}, asym	2
1620	COO^{-}_{uni}, asym	3
2170	C≡C	
	QD Films - post treatment	
2197	C≡C	-
2400-3600	-OH (H-bonded)	-
3050	CH	-
3260		-
3480		-
	As-cast films	
1398	COO^{-}_{bridge}, sym	1b
1415	COO^{-}_{bridge}, sym	1a
1524	COO^{-}_{bridge}, asym	1a
1547	COO^{-}_{bridge}, asym	1b
2851	C-H	-
2900	C-H	-
2923	C-H	-
2955	C-H	-
3004	C-H	-

5. X-ray Scattering (GISAXS and GIWAXS)

Experimental Methods

Grazing Incidence X-Ray Scattering

Both grazing incidence wide-angle X-ray scattering (GIWAXS) and small-angle X-ray scattering (GISAXS) were performed at 13.5 keV at the complex materials scattering beam line, 11-BM, at NSLS-II. Sample-to-detector distance and beam center were determined from a silver behenate standard. GIWAXS was recorded with a Pilatus 800K detector at a nominal distance of 257 mm and an angle of incidence (AOI) of 0.15 °, GISAXS with a Pilatus 2M detector at a nominal distance of 1993 mm and an angle of incidence of 0.20 °. Both AOI are above the critical angle (0.132 °) of the Si substrate. For GISAXS, the q_z center was displaced .0085 $Å^{-1}$ above the transmission center to approximate the refractive index corrections to I(q) in the region of the first prominent feature at $q_r \approx 0.1$ $Å^{-1}$. Data was analyzed in the Nika software package[†].[12] Detector images were tiled in the vertical direction to remove horizontal detector artifacts. Pole figures are symmetrized about $\chi=0$ to remove vertical detector artifacts.

Spectroscopic Ellipsometry

SE was performed with a JA Woollam CO M2000-D spectroscopic instrument, nominally spanning (200 to 1700) nm. Analysis was performed in CompleteEase software using a Kramer-Kronig consistent b-spline model to describe the dielectric function of the film.

[†] Certain commercial equipment, instruments, or materials are identified in this paper in order to specify the experimental procedure adequately. Such identification is not intended to imply recommendation or endorsement by NIST, nor is it intended to imply that the materials or equipment identified are necessarily the best available for the purpose.

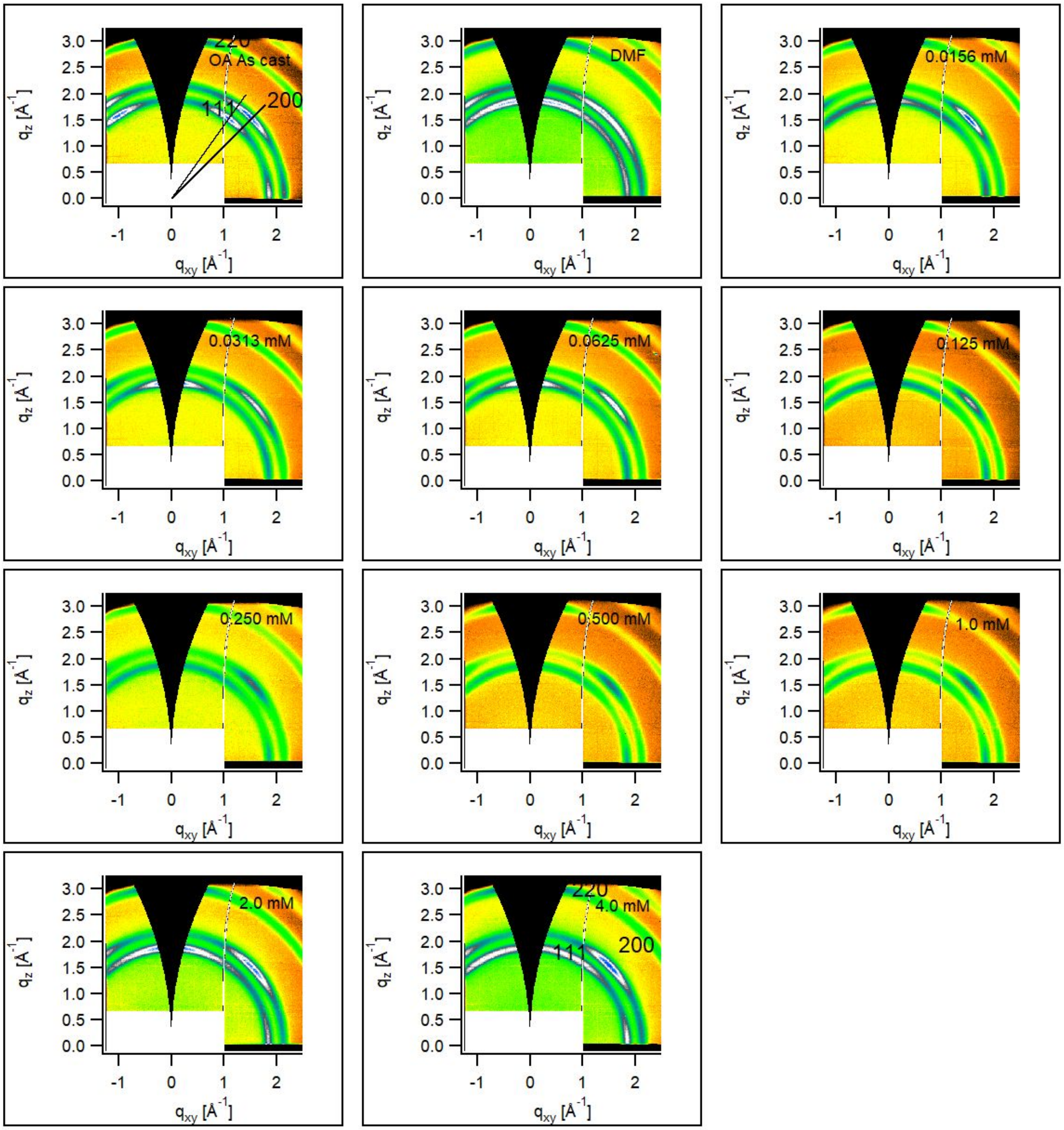

Figure S14. GIWAXS images for films on Si substrates. Large white region of low q_z is due to a missing module in the detector, enabling simultaneous SAXS. Narrow white feature at $\approx q_{xy}$ 1 Å^{-1} is a detector artifact.

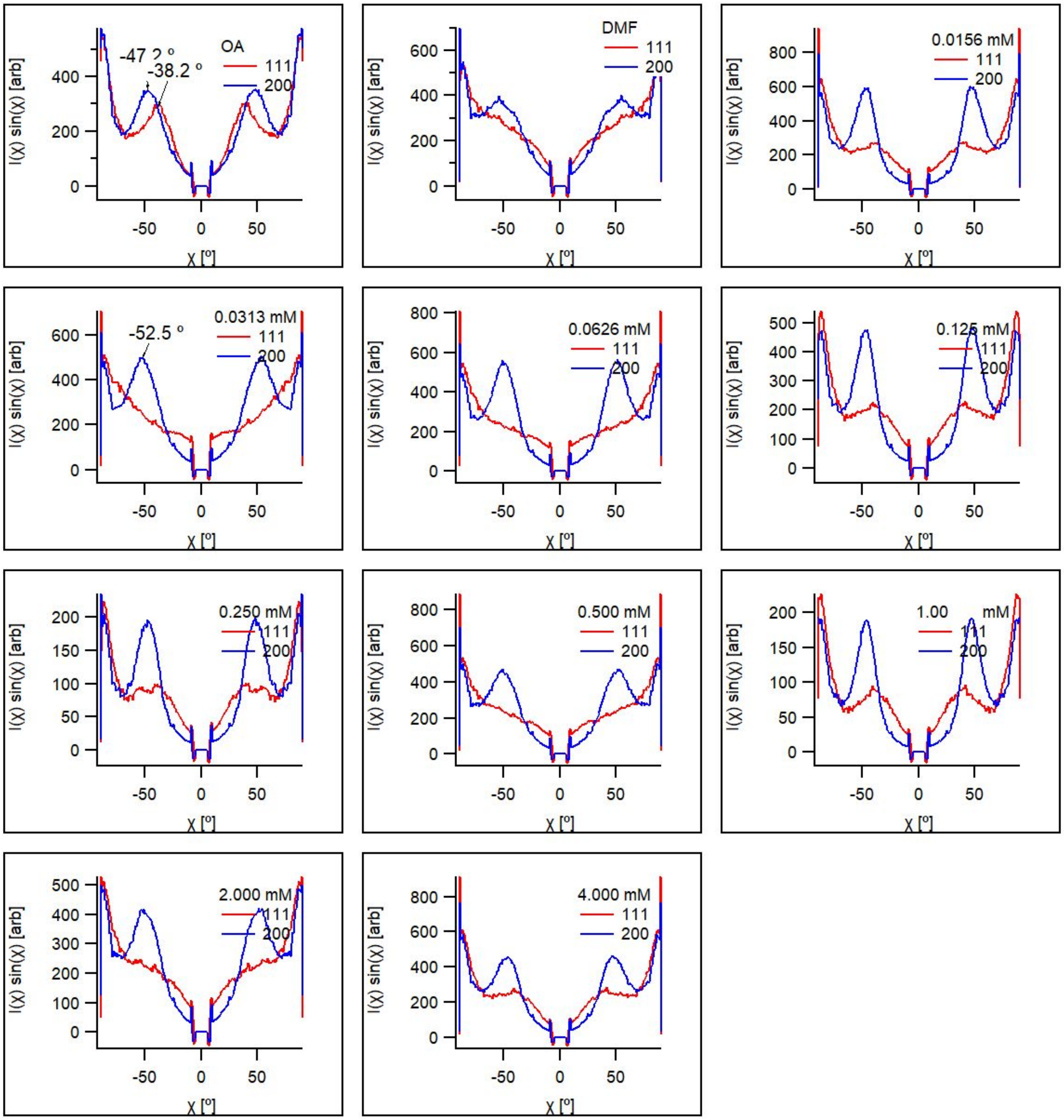

Figure S15. Sin(χ) weighted Pole figures for [111] and [200] features in GIWAXS of Figure S5.

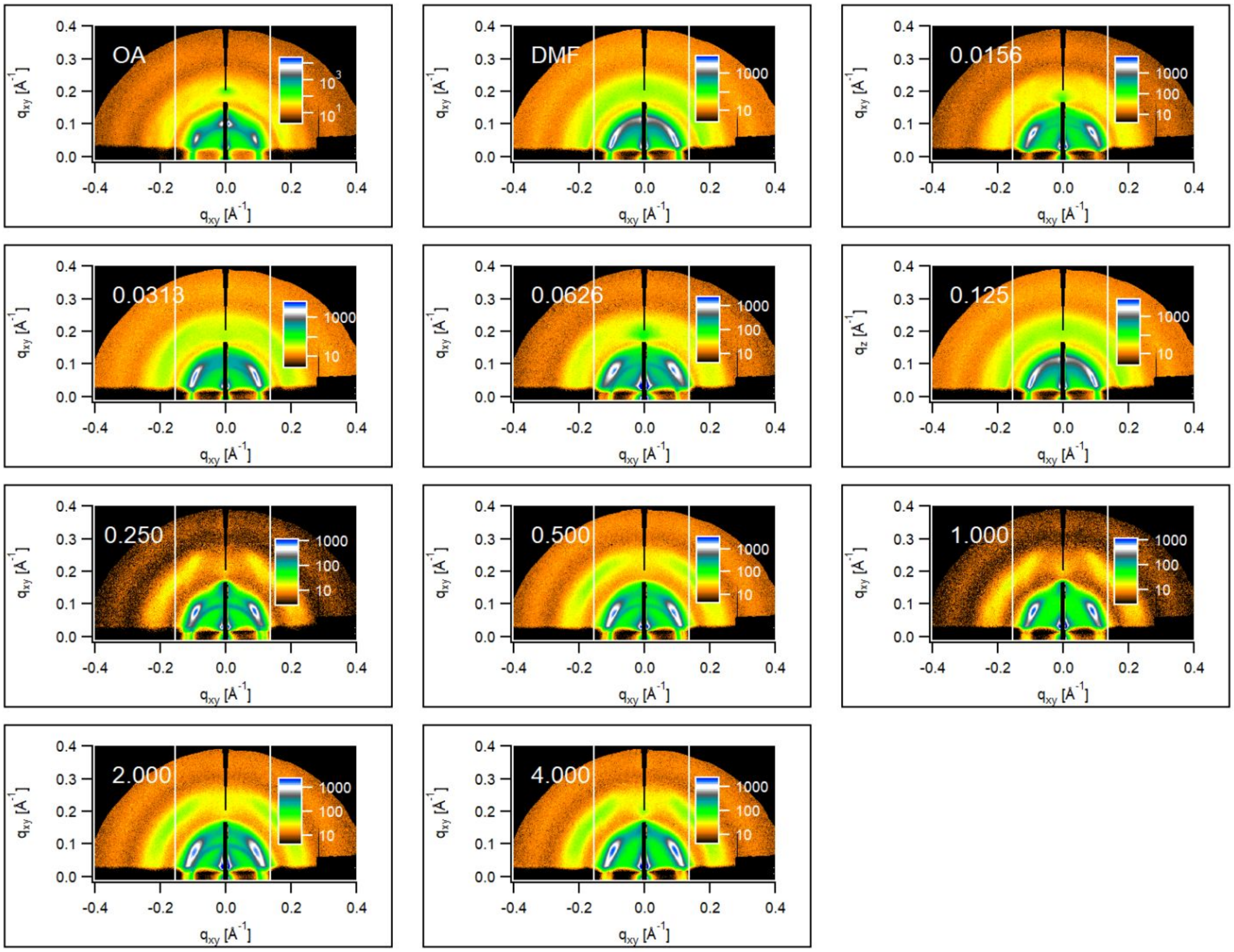

Figure S16. GISAXS images for films on Si Substrates.

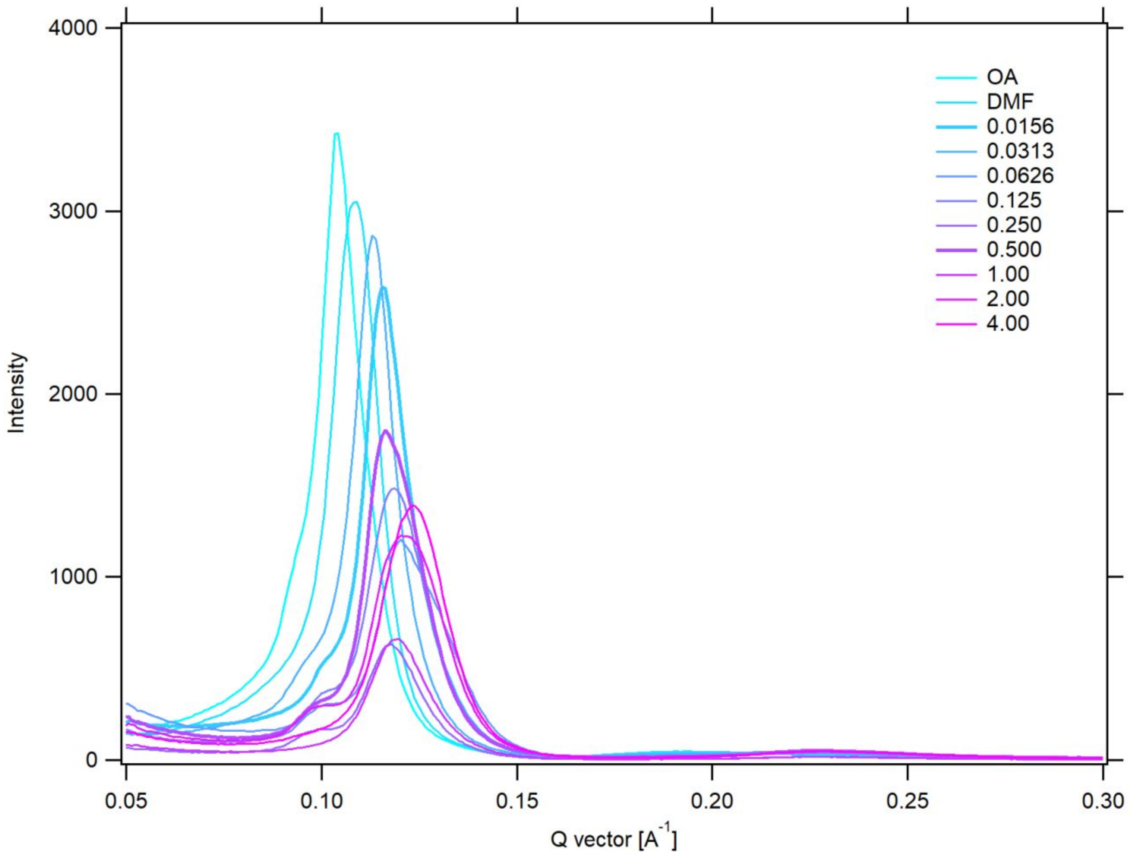

Figure S17. GISAXS sector (±10 °) cut at χ=50 ° through data in Figure S7. Data for the OA sample was taken at χ=57 °

Table S3. Location of peak GISAXS scattering along the bcc [110] &/or fcc [111] and particle-particle (p-p) spacing estimated from 7.69/q_{peak}.

Concentration [mM]	q_{peak} [Å^{-1}] *	p-p distance [Å]	Film thickness [nm]**
Oleic acid	0.104±0.002	74±1	186±2
DMF control	0.109±0.002	71±1	175±1
0.0156	0.115±0.002	67±1	144±4
0.0313	0.113±0.002	68±1	147±1
0.0626	0.120±0.002	64±1	119±1
0.125	0.119±0.002	65±1	138±1
0.250	0.118±0.002	65±1	138±2
0.500	0.116±0.002	66±1	135±1
1.00	0.119±0.002	65±1	137.4±0.4
2.00	0.120±0.002	64±1	120±1
4.00	0.124±0.002	62±1	111.8±0.3

* Uncertainty is estimate of the standard error based on both peak location and refractive index correction.

** Uncertainty is estimate of the standard error based on multiple spots on one film.

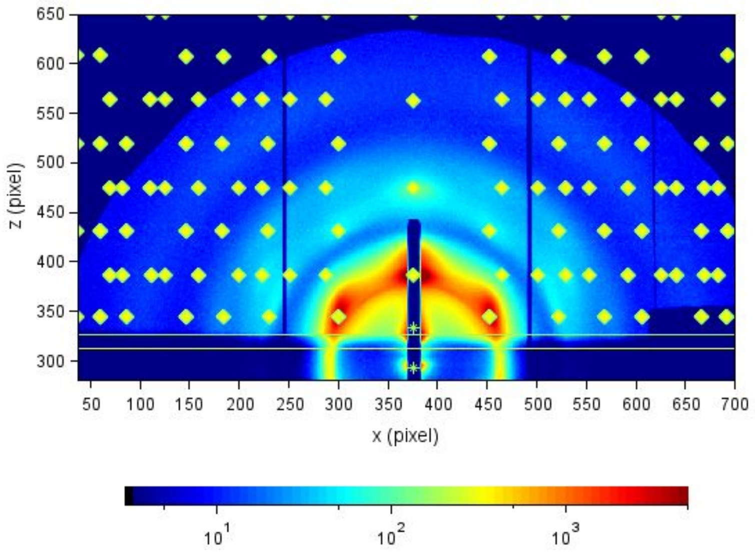

Figure S18. Indexing OA sample to bcc [110] with lattice constant a = 8.54 nm. Indexing performed with indexGIXS.[14]

bct [100]	fcc [100]
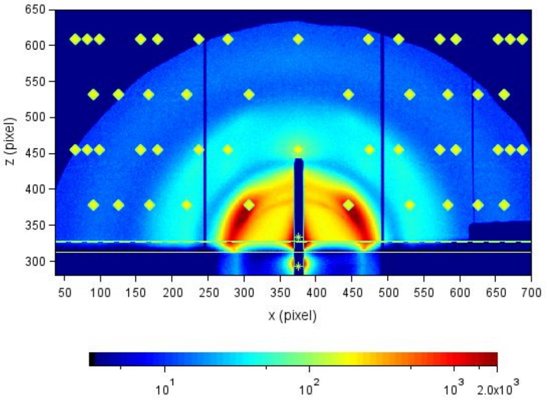	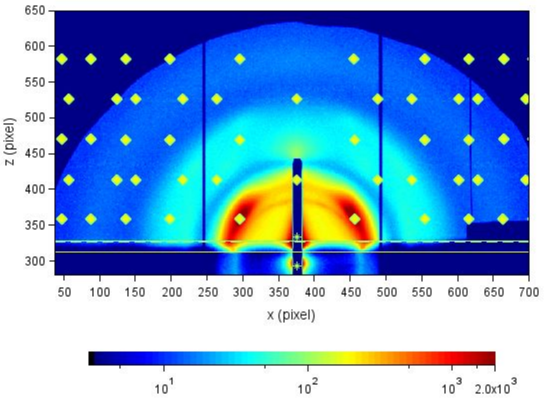
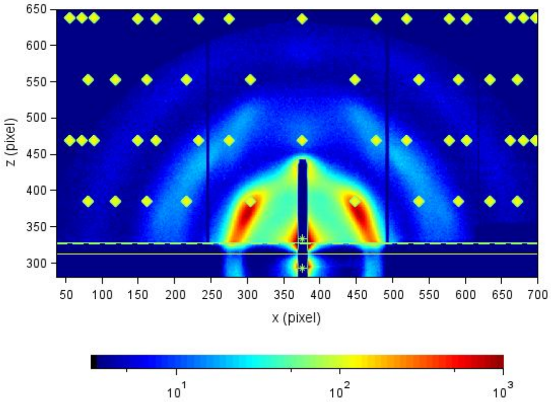	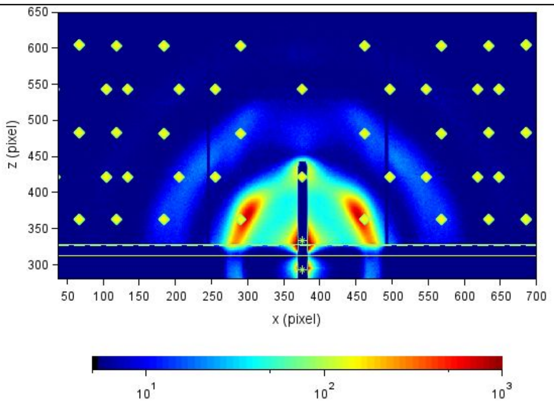

Figure S19. Possible indexing of superlattice peaks to bct [100] (with a compressed a axis) or fcc [100]. Top 0.0156 mM, Bottom 1.00 mM. Indexing performed with indexGIXS.[iii]

6. Transient Absorption Spectra

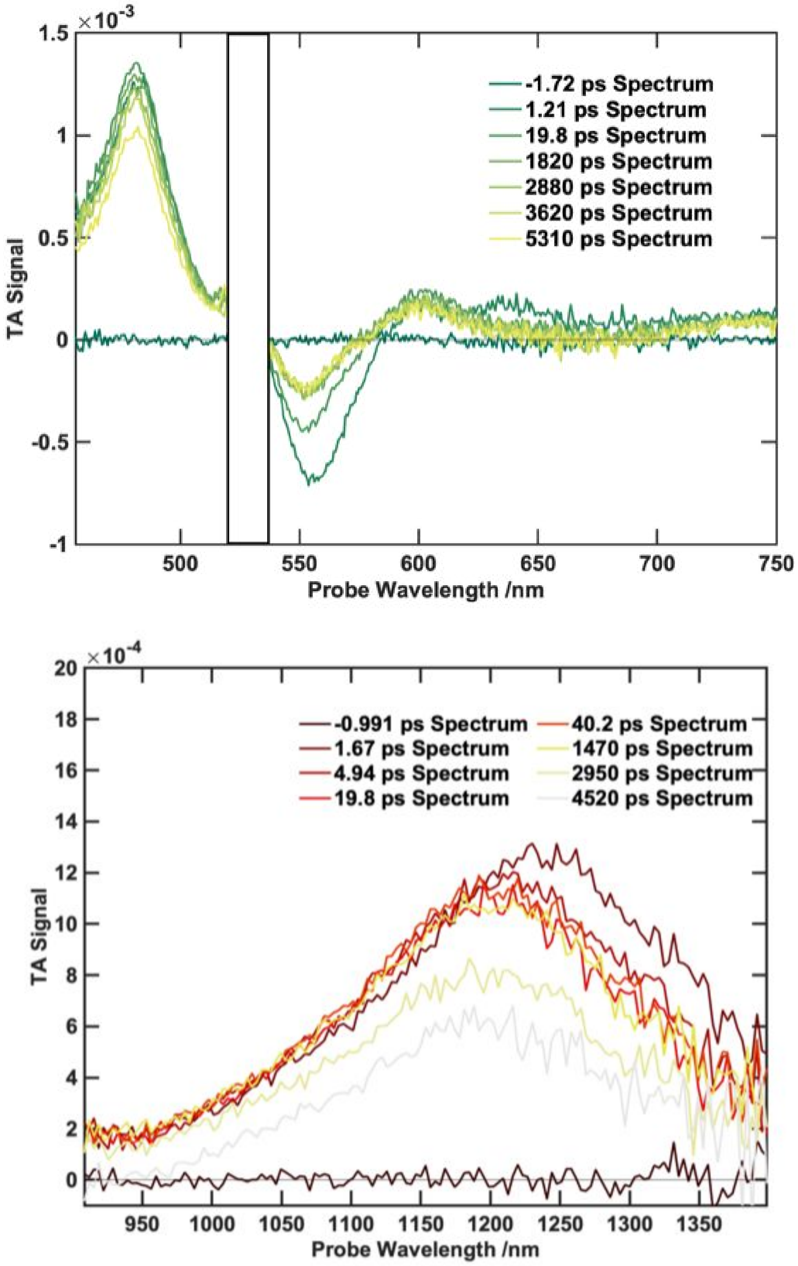

Figure S20. Visible (top) and NIR (bottom) TA spectral slices of a neat solution of $Tc(Ac\text{-}COOH)_2$ in THF, pumped at 520 nm, and 150 nJ excitation power.

7. Ligand Synthesis and Characterization

Materials

5,12-napthacenequinone was purchased from TCI, trimethylsilylacetylene from Oakwood Chemical, and tin(II) chloride was purchased from sigma-aldrich and used after purification via re-precipitation with acetic anhydride. THF was freshly distilled over sodium metal and stored over 3Å molecular sieves before use. ACS-grade DMSO was purchased from Thermo Fisher and stored over 3Å molecular sieves before use.

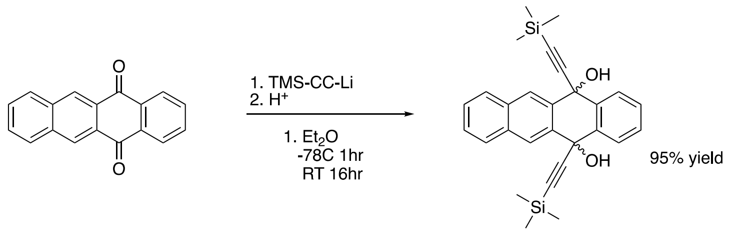

Synthesis of 5,12-Bis[(trimethylsilyl)ethynyl]-tetracene-5,12-diol

To a 100 mL round bottom was added 5,12-napthacenequinone (1g, 3.84mmol) and placed under high vacuum for 1 hour to make the reagent as moisture free as possible. While this is under vacuum, a separate 50mL pear shaped flask equipped with a Teflon magnetic stir bar was capped with a septum and then cycled through N_2/high vacuum three times. Approximately 25mL of freshly distilled diethyl ether was cannulated into the pear shaped flask. This was followed by the addition of trimethylsilyl acetylene (1.132g, 11.5*10^-2 mmol) via a vacuum dried 1mL gas-tight glass syringe. The pear flask was then placed in a dry ice/IPA bath and allowed to cool to -78 C. Once cooled, n-butyl lithium (4.38 mL from a 2.5M solution in hexanes, 11.5 mmol) was added via a vacuum dried 10mL gas-tight glass syringe dropwise. The reaction was allowed to stir at -78 C for 10 minutes before being moved to brine-ice bath to stir at -10C for an additional 40 minutes. The n-butyl lithium trimethyl silyl acetylene reaction was then cannulated over the 5,12-napthacene quinone under nitrogen dropwise. The reaction is left to stir in the 100mL round bottom overnight at room temperature covered in foil.

The next day the solution is clear and ruby red in color (sometimes the reaction maintains the forest green color overnight). The reaction was quenched with approximately 25mL of a saturated ammonium chloride

solution. The organics were extracted with DCM, washed with 30mL of water two times, then 30mL of a brine solution, and finally dried with MgSO4. The solvent was filtered then removed in vacuo to produce a relatively pure mixture of the cis and trans isomers as green oil. These isomers can be then isolated via 1:1 DCM:Hexanes to produce an off white solid. 1.63 grams (95%) ^{1}HNMR (300 MHz, ppm, $CDCl_3$): 0.19 (m, 18 H), 3.44 (s, 2 H), 7.47-7.51 (m, 2 H), 7.53-7.56(m, 2 H), 7.91-7.95 (m, 2), 8.11-8.14 (m, 2 H), 8.56 (s, 1 H). ^{13}C NMR (400 MHz, ppm, CDCl3) δ 0.19, 69.34, 93.21, 107.00, 125.96, 126,25, 126.97, 128.32, 129.21, 133.19, 136.13, 138.51.

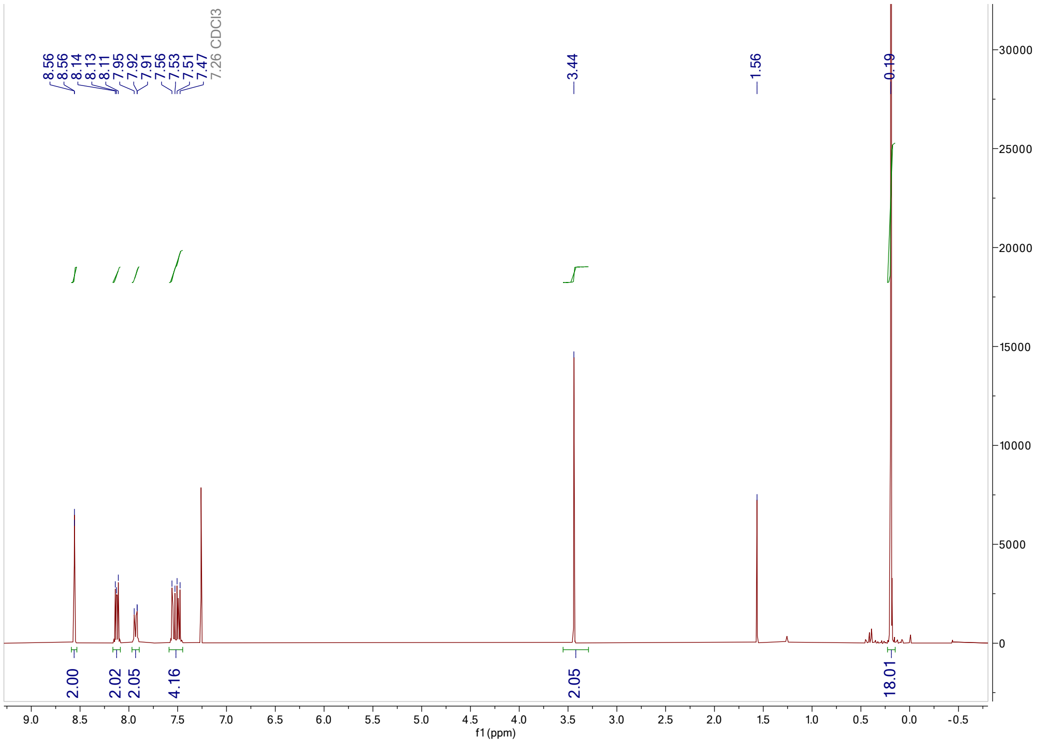

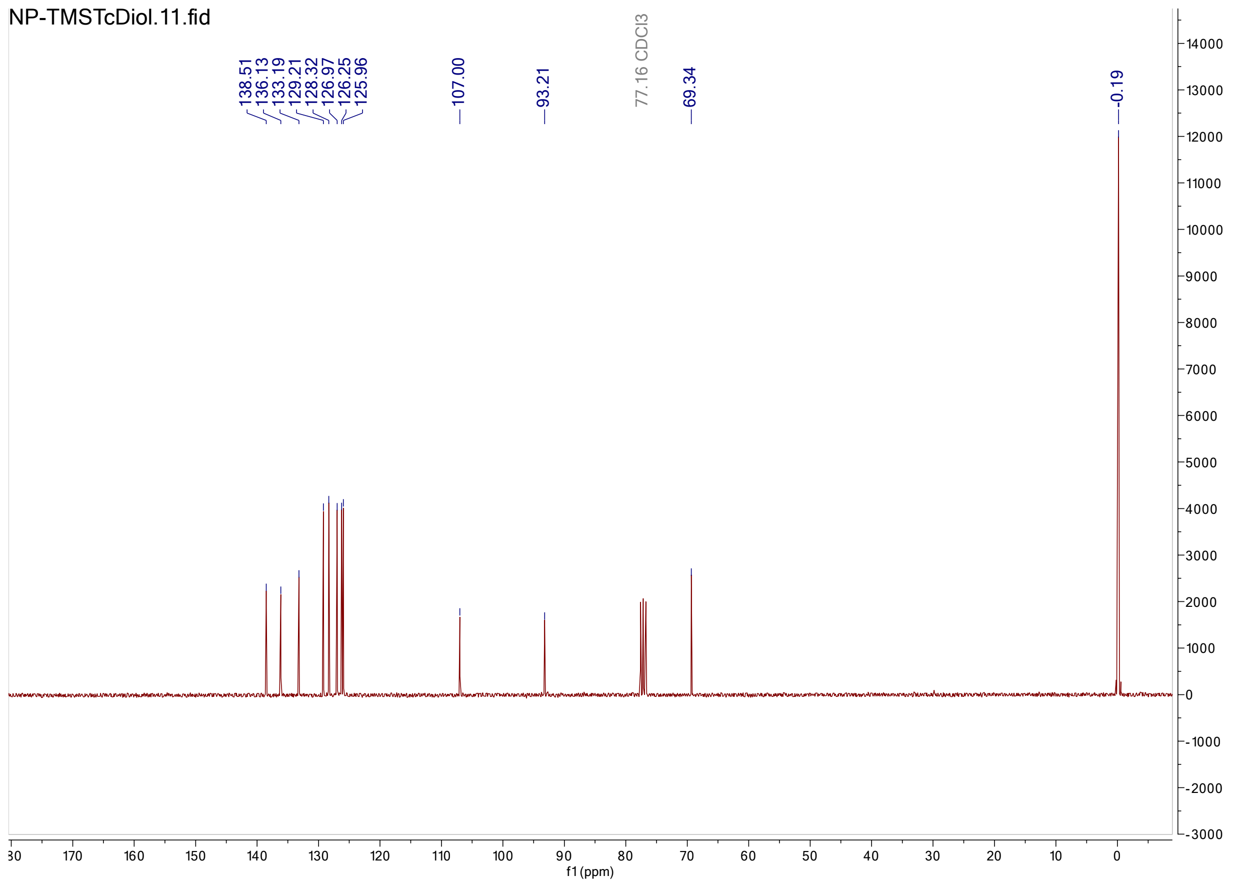

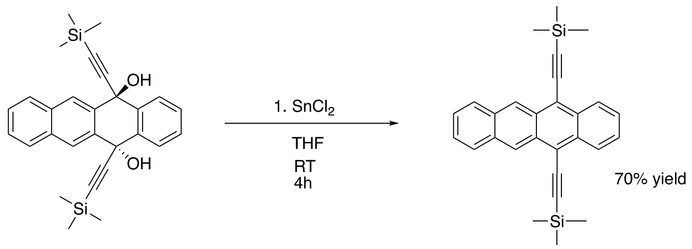

Synthesis of 5,12-Bis[(trimethylsilyl)ethynyl]tetracene

5,12-Bis[(trimethylsilyl)ethynyl]-tetracene-5,12-diol (0.718g, 1.58 mmol) was transferred to an oven dried 25mL round bottom flask equipped with a medium stir bar and dissolved in approximately 15mL of freshly distilled THF. The solution was then sparged with N2 for five minutes before adding tin(II) chloride (1.20g, 6.32 mmol) as a solid in one portion. This changes the color of the solution from a clear tan to red almost instantaneously. The reaction flask is covered in foil and allowed to stir in the dark at room temperature for 4 hours. After 4 hours the THF is removed in vacuo. The red solid is then redissolved in chloroform and pushed through a small silica plug with chloroform to remove residual tin(II) chloride. The chloroform is then removed in vacuo. This red solid can then be purified via a silica

column with n-hexanes mobile phase to produce TMS-tetracene in as a ruby red colored solid. This material is recrystallized from chloroform layered with methanol (1:4) to yield red-needle like crystals. 464mg. (70% yield). ^{1}HNMR (300 MHz, ppm, $CDCl_3$) 0.48 (m, 18 H), 7.46-7.49(m, 2 H), 7.53-7.56 (m, 2H), 8.06-8.10 (m, 2), 8.55-8.59 (m, 2 H), 9.20 (s, 1 H). ^{13}C NMR (400 MHz, ppm, CDCl3) δ 0.42, 102.24, 109.32, 118.45, 126.14, 126.25, 126.86, 127.51, 128.75, 130.17, 132.30, 132.65

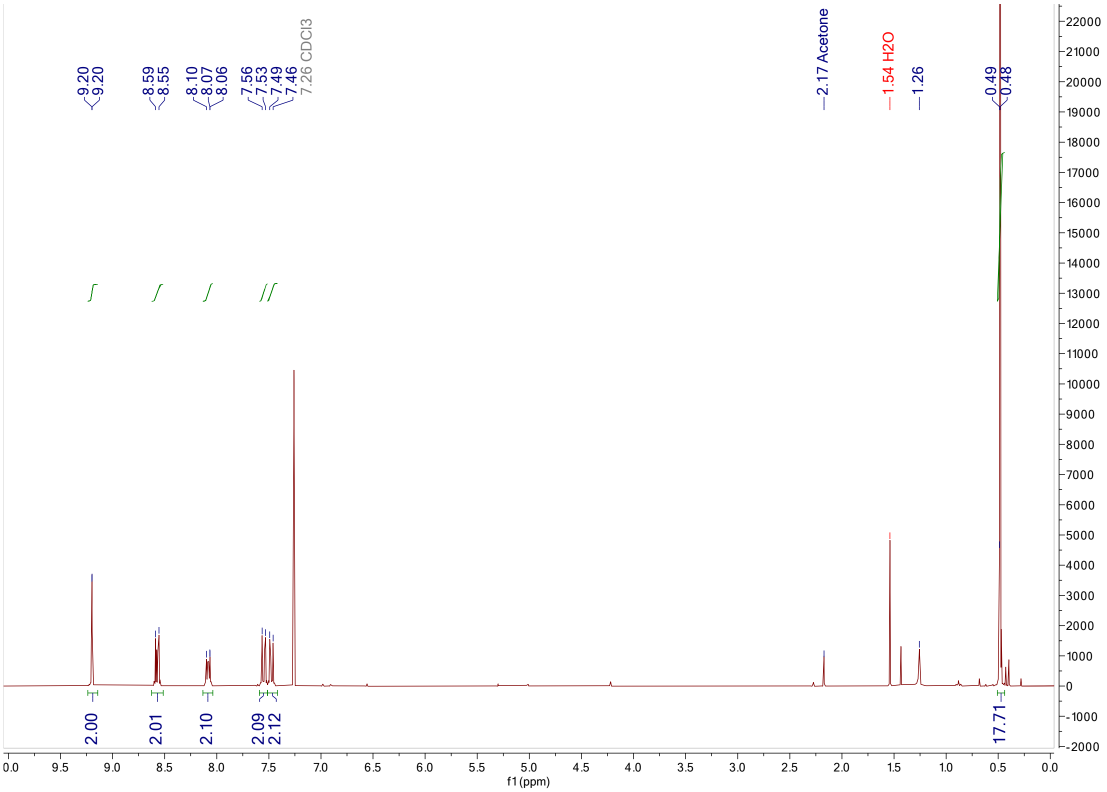

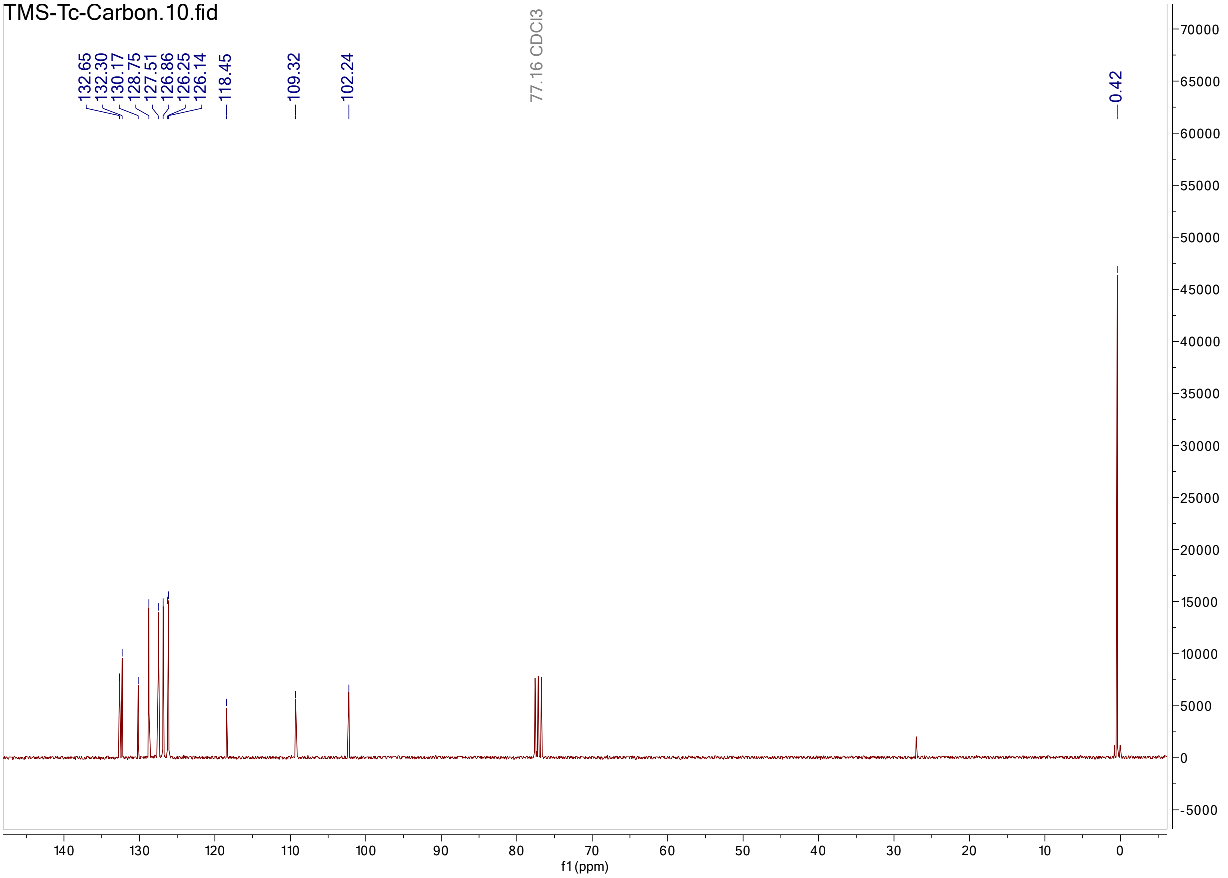

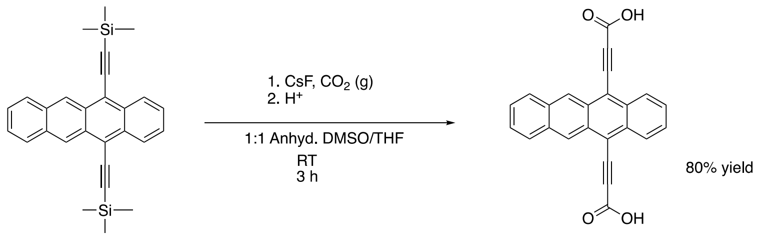

Synthesis of 5,12-Bis[(carboxylicacid)ethynyl]tetracene

To an oven dried 10 mL round bottom flask equipped with small stir bar was added CsF (0.063g, 0.414 mmol) within a glovebox under an N_2 atmosphere. The round bottom was capped with a septum and pumped out of the glovebox and immediately placed under high vacuum. It was then cycled through N_2/High vacuum three times before 1.5mL of anhydrous DMSO was added via 2.5 mL vacuum dried glass syringe followed by 0.5 mL of freshly distilled THF to aid in the TMS Tc solubility. The opaque solution was then sparged with CO_2 for 30 minutes before uncapping and adding TMS-tetracene (0.116g,

0.276 mmol) quickly as a solid in one portion. The round bottom was quickly recapped and the solution was continued to be sparged with CO_2. The solution quickly went from bright red to a wine red solution. After 3.5 hours, approximately 2mL of a 5% KOH(aq) solution was added to the reaction dropwise which turns the solution dark red in color. The crude solution was then poured into a Erlenmeyer flask containing 10mL of 5% KOH solution and 10mL of ethyl acetate. The biphasic solution is swirled then poured into a separatory funnel. The aqueous layer is collected and the organics removed. The aqueous layer is washed with ethyl acetate until the organic layers are no longer colored. The collective aqueous layers are acidified with a 1M HCl (aq) solution dropwise until a blue precipitate forms, approximately when the aqueous solution reaches a pH of 2. 30 mL of diethyl ether is then added to the separatory funnel to dissolve the blue precipitate forming a bright red organic layer. The organic layer is then collected, washed with 30 mL of water, then 30mL brine, and finally dried with MgSO4. The diethyl ether is removed in vacuo, leaving behind bis-carboxylic acid ethynyl tetracene (TcAcCOOH) as a blue solid. ~72mg. (80% yield). ^{1}HNMR (300 MHz, ppm, $CDCl_3$) 7.61-7.64(m, 2 H), 7.77-7.81 (m, 2H), 8.22-8.25 (m, 2), 8.46-8.49 (m, 2 H), 9.13 (s, 1 H). ^{13}C NMR (400 MHz, ppm, d^6-DMSO) δ 80.14, 95.73, 116.30, 125.27, 126.31, 127.39, 128.29, 128.69, 129.27, 132,34, 132.70, 154.23.

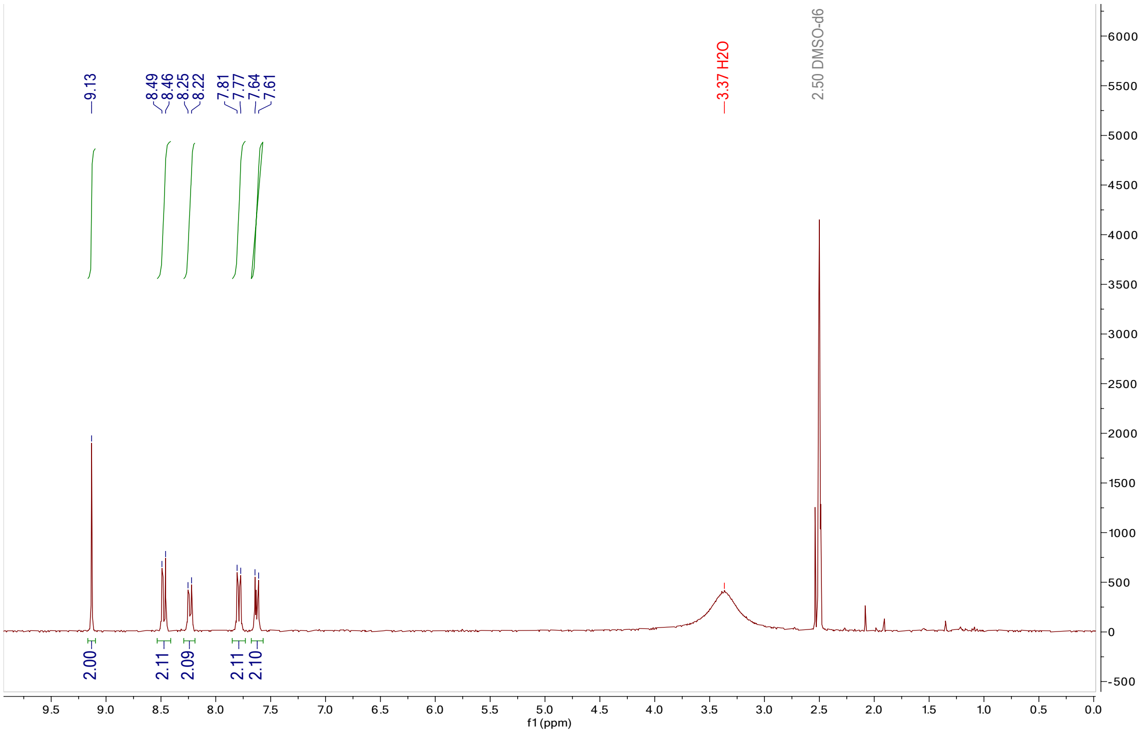

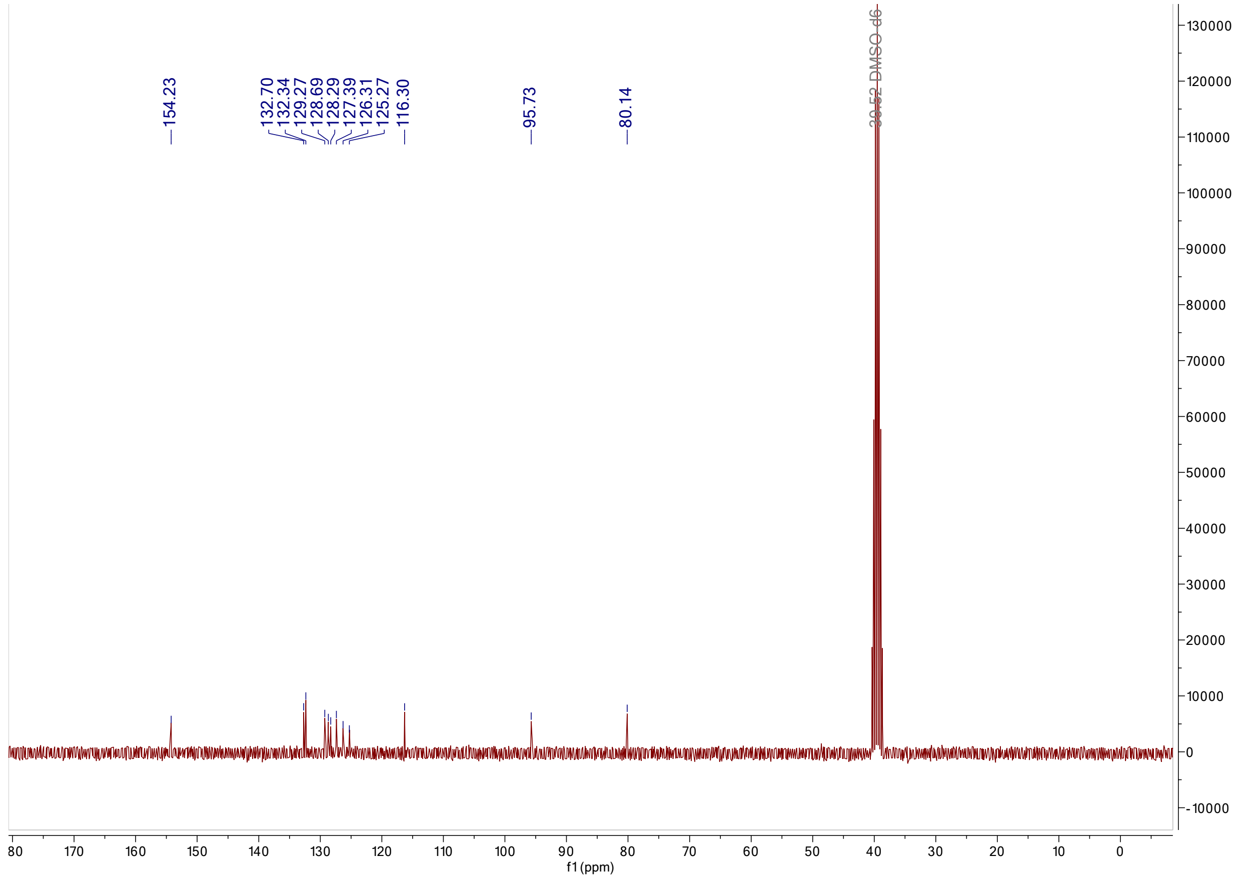

154.23
132.70
132.34
129.27
128.69
128.29
127.39
126.31
125.27
116.30
95.73
80.14
39.52 DMSO-d6
f1 (ppm)

References

1a. Kresse, G. & Furthmüller, J. Efficient iterative schemes for *ab initio* total-energy calculations using a plane-wave basis set. *Phys Rev B* **54**, 11169–11186 (1996).
1b. Kresse, G. & Furthmüller, J. Efficiency of ab-initio total energy calculations for metals and semiconductors using a plane-wave basis set. Comput Mater Sci 6, 15–50 (1996).
2. Perdew, J. P., Burke, K. & Ernzerhof, M. Generalized Gradient Approximation Made Simple. Phys Rev Lett 77, 3865–3868 (1996).
3. Grimme, S., Ehrlich, S. & Goerigk, L. Effect of the damping function in dispersion corrected density functional theory. J Comput Chem 32, (2011).
4. Kresse, G. & Joubert, D. From ultrasoft pseudopotentials to the projector augmented-wave method. Phys Rev B 59, (1999).
5. Xiaomin He, Jichao Hu, Xue Tian, Electronic characteristics of PbS quantum dots passivated by halides on different surfaces, Applied Surface Science, 568, 2021, 150736.
6. J. Muscat, J.D. Gale, First principles studies of the surface of galena PbS, Geochimica et Cosmochimica Acta, 67, Issue 5, 2003, 799-805.
7. Madelung, O., Rössler, U., Schulz, M. Lead sulfide (PbS) crystal structure, lattice parameters, thermal expansion. In *Non-Tetrahedrally Bonded Elements and Binary Compounds I*, Vol. 41C; Springer-Verlag Berlin Heidelberg, 1998
8. Monkhorst, H. J. & Pack, J. D. Special points for Brillouin-zone integrations. Phys Rev B 13, (1976).
9. Frederiksen, T., Paulsson, M., Brandbyge, M. & Jauho, A.-P. Inelastic transport theory from first principles: Methodology and application to nanoscale devices. Phys Rev B 75, 205413 (2007).
10. Deshlahra, P., Conway, J., Wolf, E.E., Schneider, W.F. Influence of Dipole–Dipole Interactions on Coverage-Dependent Adsorption: CO and NO on Pt(111) *Langmuir* 2012, 28, 22, 8408–8417, 2012
11. Wilson Jr, E. B., Decius, J. G., & Cross, P. G. Molecular vibrations. American Journal of Physics, 23(8), 550-550. (1955).
12. Ilavsky, J. Nika: Software for Two-Dimensional Data Reduction. *J Appl Cryst* **2012**, *45*, 324–328.
13. Smilgies D-M, Li R. "indexGIXS – software for visualizing and interactive indexing of grazing-incidence scattering data," ChemRxiv. Cambridge: Cambridge Open Engage; 2021 (accessed May 9th, 2022).
